# Synthesis of *N*-(4-chlorophenyl) substituted pyrano[2,3-c]pyrazoles enabling PKBβ/AKT2 inhibitory and *in vitro* anti-glioma activity

**DOI:** 10.1080/07853890.2022.2123559

**Published:** 2022-09-18

**Authors:** Ruturajsinh M. Vala, Vasudha Tandon, Lynden G. Nicely, Luxia Guo, Yanlong Gu, Sourav Banerjee, Hitendra M. Patel

**Affiliations:** a Department of Chemistry, Sardar Patel University, Vallabh Vidyanagar, India; b Department of Cellular & Systems Medicine, School of Medicine, University of Dundee, Dundee, UK; c Key Laboratory of Material Chemistry for Energy Conversion and Storage, Ministry of Education, Hubei, Key Laboratory of Material Chemistry and Service Failure, School of Chemistry and Chemical Engineering, Huazhong University of Science and Technology, Wuhan, China

**Keywords:** Pyrano[2,3-c]pyrazole, kinase inhibitor, neurosphere, anti-glioma, stem cells

## Abstract

A series of *N*-(4-chlorophenyl) substituted pyrano[2,3-c]pyrazoles was synthesised and screened for their potential to inhibit kinases and exhibit anticancer activity against primary patient-derived glioblastoma 2D cells and 3D neurospheres. A collection of 10 compounds was evaluated against glioma cell lines, with compound **4j** exhibiting promising glioma growth inhibitory properties. Compound **4j** was screened against 139 purified kinases and exhibited low micromolar activity against kinase AKT2/PKBβ. AKT signalling is one of the main oncogenic pathways in glioma and is often targeted for novel therapeutics. Indeed, AKT2 levels correlated with glioma malignancy and poorer patient survival. Compound **4j** inhibited the 3D neurosphere formation in primary patient-derived glioma stem cells and exhibited potent EC_50_ against glioblastoma cell lines. Although exhibiting potency against glioma cells, **4j** exhibited significantly less cytotoxicity against non-cancerous cells even at fourfold–fivefold the concentration. Herein we establish a novel biochemical kinase inhibitory function for *N*-(4-chlorophenyl) substituted pyrano[2,3-c]pyrazoles and further report their anti-glioma activity *in vitro* for the first time.

**KEY MESSAGE**
- Anti-glioma pyrano[2,3-c]pyrazole **4j** inhibited the 3D neurosphere formation in primary patient-derived glioma stem cells. **4j** also displayed PKBβ/AKT2 inhibitory activity. **4j** is nontoxic towards non-cancerous cells.

## 1. Introduction

Protein kinases have diverse roles in cellular physiology [1]. Over 520 protein kinases have been reported *via* human kinome tree in 2002 [2], while many new kinases have been identified since [3]. The oncogenic roles of protein kinases have long been targeted for therapeutic benefits [4,5], while a few kinases exhibit tumour suppressor properties as well [4,6]. Although over 70 kinase inhibitors have been approved for clinical use [5], very few options are available for patients with grade IV glioma or glioblastoma where median survival remains at 12–18 months from initial diagnosis [7]. Gliomas are the most common central nervous system cancer arising from glial cells within the brain. Pathologically sub classifying the tumour into IDH1 mutation and chromosome 1p/19q co-deletion status can improve patients’ therapeutic options [8,9]. Although patients with IDH1 mutant and 1p/19q co-deleted glioma tumours exhibit improved survival and outcome [9], many eventually develop resistance and refractory glioma. Chromosome 19q, in fact, has a pro-tumorigenic resident kinase called AKT2/PKBβ [10]. AKT is an established oncogenic kinase with at least three mammalian isoforms AKT1/PKBα, AKT2/PKBβ, and AKT3/PKBγ of which AKT2 plays major pro-oncogenic roles in cancers, including glioma [10].

**Figure 1.**
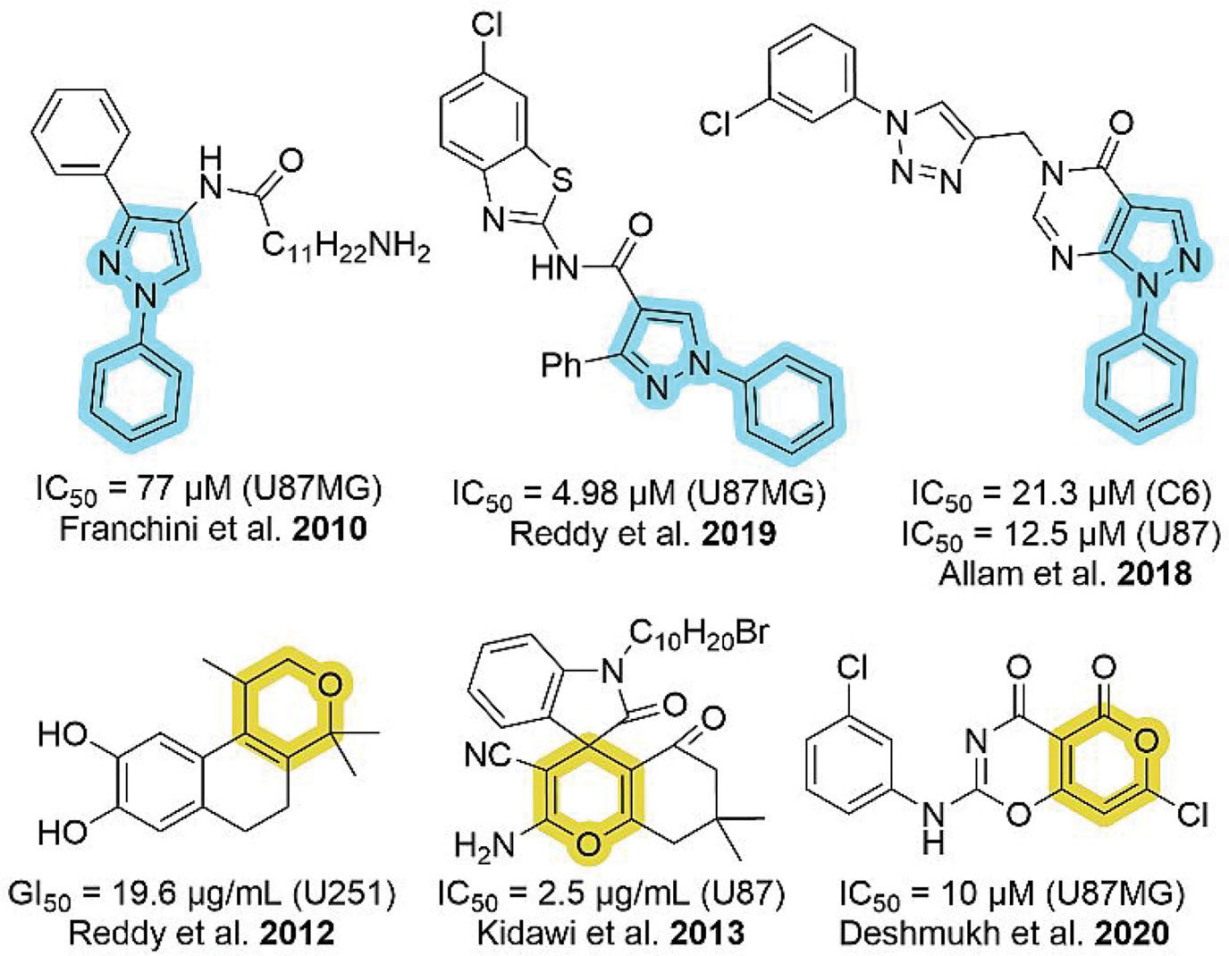
Cytotoxic activities of *N*-phenyl substituted pyrazole and pyran moieties against different glioma cell lines.

As shown in Figure 1, *N*-phenyl substituted pyrazole and pyran containing heterocycles displayed good anti-glioma activity as shown in U87MG, C6, U87, and U251 cells [11–16]. In addition, three heterocycles out of six contain the chlorine group. So, we designed heterocycles containing *N*-phenyl substituted pyrazole and pyran moieties with chlorine group to test for anti-glioma activity. Hence, we synthesised *N*-(4-chlorophenyl) substituted pyrano[2,3-c]pyrazoles.

**Scheme 1.**
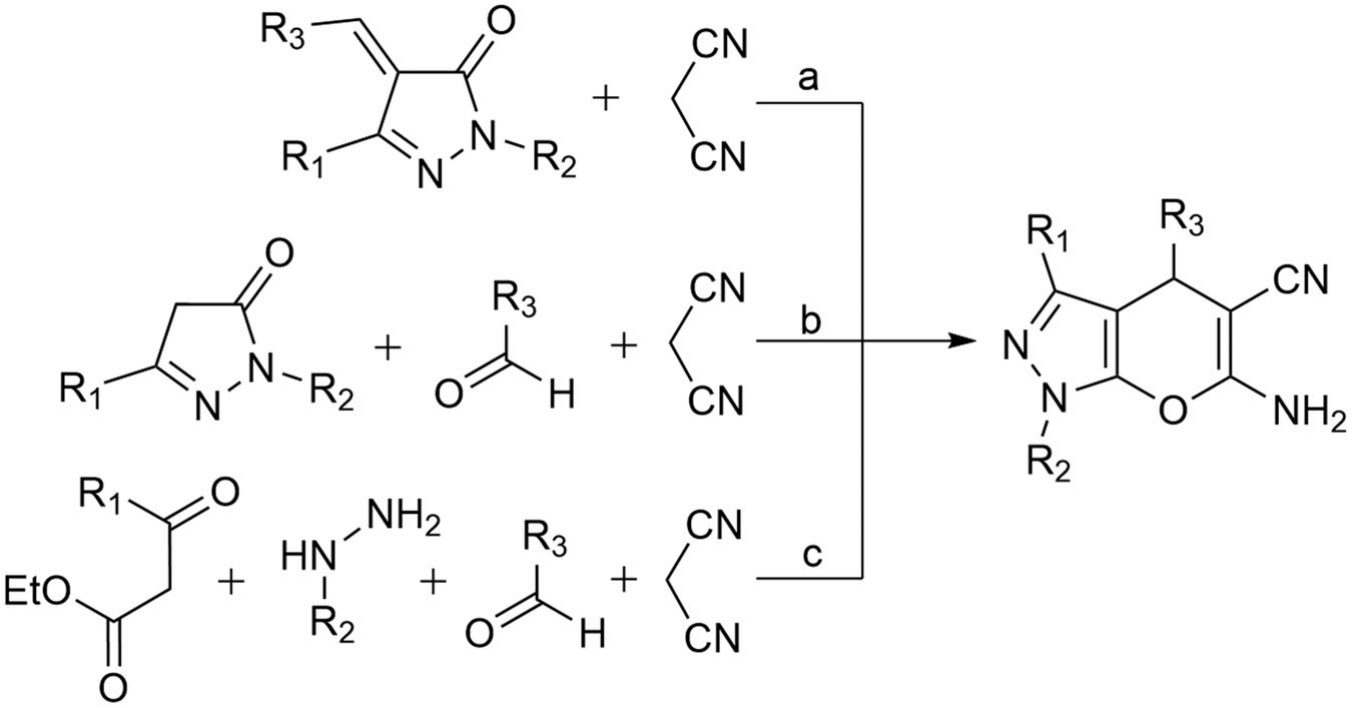
Two and multicomponent synthesis of pyrano[2,3-C]pyrazoles. Reaction type: (a) two-component, (b) three-component, and (c) four-component.

Biologically potent pyrano[2,3-c]pyrazoles synthesised by two-component, three-component, and four-component synthesises have been reported previously (Scheme 1) [17]. A two-component reaction was reported wherein 4-aryliden-pyrazol-5-one and malononitrile were reacted together to produce pyrano[2,3-c]pyrazoles (Scheme 1(a)) [18]. Three-component synthesises of pyrano[2,3-c]pyrazoles were reported through a reaction of aldehyde, 5-pyrazolone, and malononitrile (Scheme 1(b)) [19–24]. Furthermore, four-component reactions of aldehyde, malononitrile, β-keto ester and hydrazine hydrate derivatives have also been reported (Scheme 1(c)) [25–29]. These reactions were catalysed using various basic catalysts [23–25,29], ionic liquid catalysts [30], and metal catalysts [22,26,31]

**Scheme 2.**
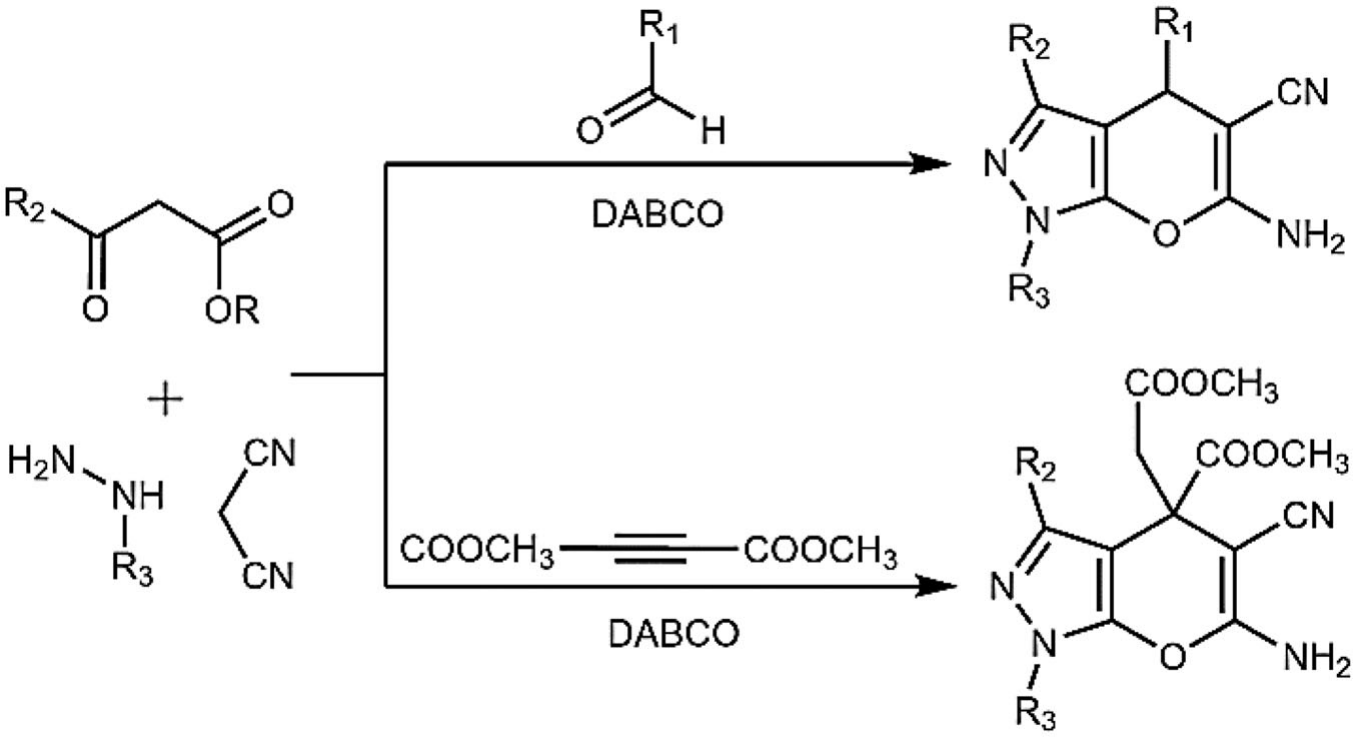
Two and multicomponent synthesis of pyrano[2,3-C]pyrazoles. Reaction type: (a) two-component, (b) three-component, and (c) four-component.

**Scheme 3.**
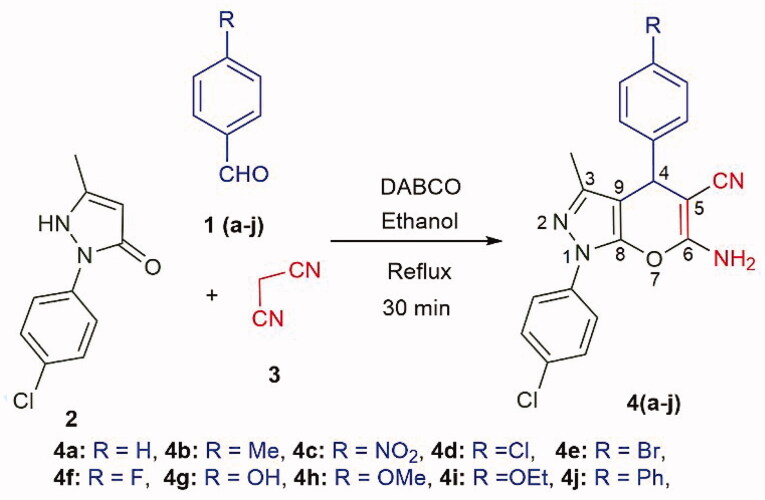
Synthesis of pyrano[2,3-c]pyrazoles by three-component reaction (with labelled atoms).

In the current work, we used DABCO as a catalyst to synthesise *N*-(4-chlorophenyl) substituted pyrano[2,3-c]pyrazoles. DABCO is a highly reactive, inexpensive, eco-friendly, nontoxic catalyst for various organic transformations [32]. As shown in Scheme 2, two types of DABCO catalysed four-component synthesis of pyrano[2,3-c]pyrazoles are available. The first method is similar to Scheme 1(c). In the second method, the aldehyde is replaced by dimethyl acetylenedicarboxylate. Both methods produce pyrano[2,3-c]pyrazoles, but substitutions at C-4 are different. In continuation of works on aldehyde based three-component reactions [33–39], in current work, we synthesised *N*-(4-chlorophenyl) substituted pyrano[2,3-c]pyrazoles by aldehyde based DABCO catalysed three-component reaction (Scheme 3). We further report **4j** as the first pyrano[2,3-c]pyrazole derivative with kinase inhibitory and anti-glioma potency from within the series. Compound **4j** inhibited AKT2/PKBβ specifically among 139 purified kinases tested and induced cell death in primary patient-derived glioma 2D cells and 3D neurospheres while being relatively nontoxic towards non-cancerous cells.

## 2. Results and discussion

### 2.1. Chemistry

We synthesised 6-amino-1,4-dihydropyrano[2,3-c]pyrazole-5-carbonitriles **4(a-j)** by three-component reaction of aldehyde **1**, 1-(4-chlorophenyl)-3-methyl-5-pyrazolone **2** and malononitrile **3** (Scheme 3). DABCO efficiently catalysed the reaction with ethanol as a reaction medium and gave moderate to excellent yield in 30 min. The synthesised compounds were characterised by ^1^H-NMR, ^13^C{^1^H}-NMR spectroscopy and HRMS analysis.

In ^1^H-NMR, the singlet peak observed at δ 1.78–1.84 ppm confirmed the presence of a methyl group of pyrazole rings. Proton present at the fourth position of pyran ring confirmed by singlet peak observed at δ 4.62–4.93 ppm. Due to eight aromatic protons, signals were observed in the region δ 6.72–8.23 ppm. In this region, the singlet peak of the amine group substituted in the pyran ring also appears at δ 7.18–7.53 ppm.

^13^C{^1^H}-NMR of **4a** show a total of 16 signal for nonequivalent carbon atoms. A signal appeared at δ 12.56 ppm due to methyl carbon substituting at the C-3 position. In comparison, C-3 carbon gave a signal at δ 143.95 ppm. The signal that appeared at δ 36.70 ppm confirmed the presence of carbon C-4. Other pyran carbons appeared at δ 58.16 (C-5), 159.34 (C-6), 145.74 (C-7a), 98.89 (C-3a) ppm. The signal of nitrile carbon appeared at δ 119.93 ppm. Eight signals that appeared in the range δ 121.37–143.48 ppm confirmed the presence of aromatic carbons. The same pattern of signals was observed in ^13^C{^1^H}-NMR of other synthesised products. Some deviation in the position of signals was observed due to the effect of a substituted functional group at the fourth position of phenyl ring substituted in pyran ring.

All synthesised pyrano[2,3-c]pyrazoles are characterised by mass spectrometry. The molecular ion peak was observed in HRMS of each synthesised compound matched with its calculated value.

### 2.2. Biology

#### 2.2.1. Compound **4j** had the most potent antiproliferative activity in GL261 cells

To determine the biological relevance of these molecules *in vitro*, EC_50_ values were conducted on all synthesised compounds in triplicates against murine glioblastoma cell line GL261. Out of the 10 molecules tested, **4j** had the lowest EC_50_ value of 20 μM (Table 1). Potent PKB inhibitor MK-2206 is a reference compound that exhibits an EC_50_ value of 10-fold lower than **4j**. Table 1 shows EC_50_ (μM) values of compounds **4a–4j** in GL261 cells *in vitro*.

**Table 1.** EC_50_ (μM) values of compounds **4a–4j** in GL261 cells *in vitro.*

Entry	Compound	R	GL261 EC_50_ (μM)^a^
1	**4a**	H	>50
2	**4b**	Me	>50
3	**4c**	NO_2_	>100
4	**4d**	Cl	>100
5	**4e**	Br	>100
6	**4f**	F	>100
7	**4g**	OH	>100
8	**4h**	OMe	>100
9	**4i**	OEt	>50
10	**4j**	Ph	20 ± 3
*Ref*	**MK-2206**	–	2 ± 0.5

^a^The EC_50_ values for antiproliferation activity were determined by a dose-response inhibition curve. All assays were performed in triplicate.

#### 2.2.2. Compound **4j** specifically inhibits AKT2/PKBβ *in vitro*

**Figure 2.**
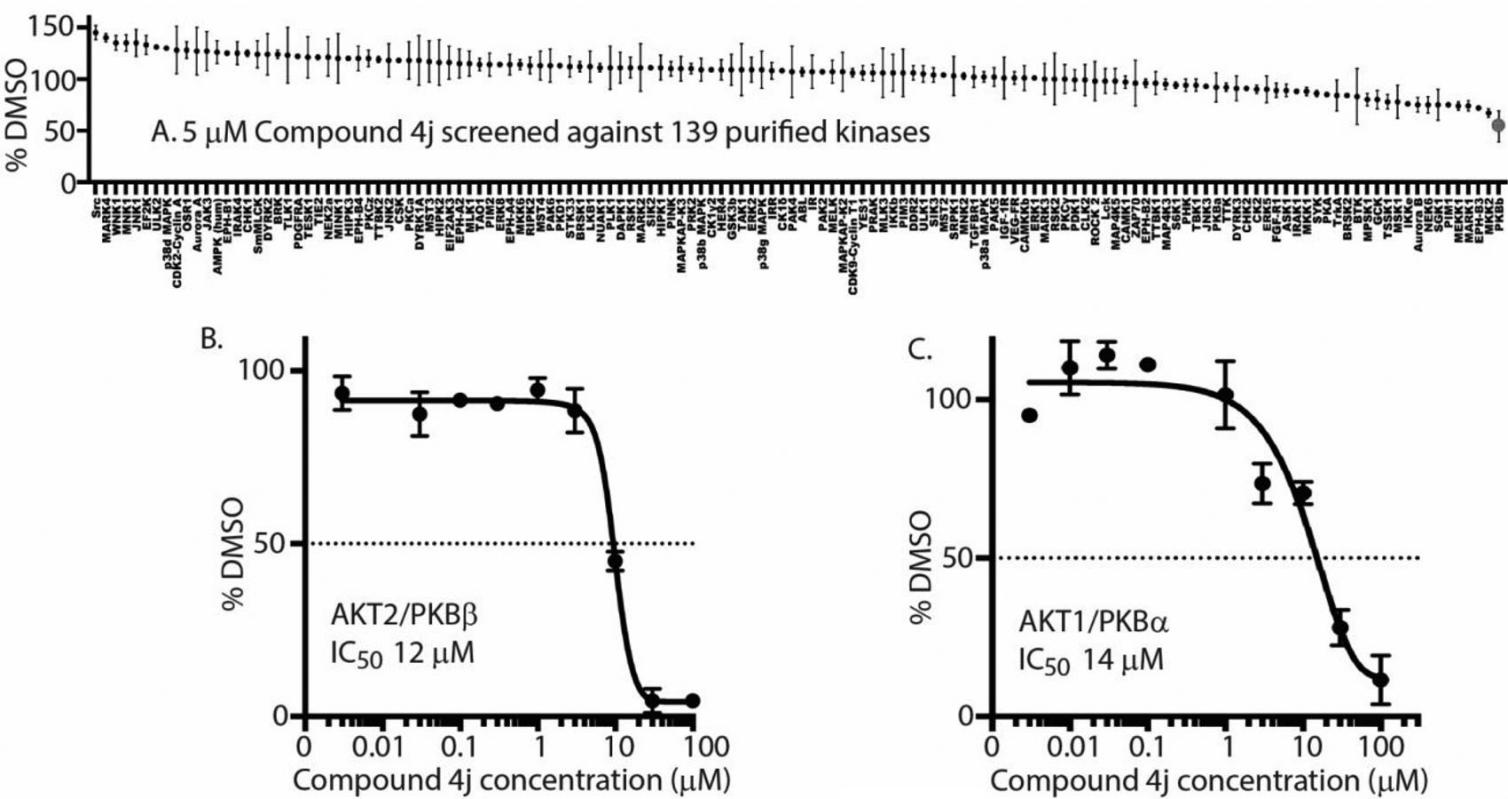
**4j** exhibits biochemical inhibitory activity against AKT2 (A) Kinase profiling of **4j** at 5 μM was carried out against the panel of 139 kinases at the International Centre for Protein Kinase Profiling (http://www.kinase-screen.mrc.ac.uk/). The IC_50_ value of **4j** was recorded *in vitro* using purified AKT2 (B) and AKT1 (C) over different **4j** concentrations.

To identify any potential kinase inhibitory activity of **4j**, 5 μM **4j** was screened over 139 purified kinases at the International Centre for Protein Kinase Profiling, University of Dundee, UK. Interestingly, **4j** significantly inhibited PKBβ/AKT2 with a high degree of specificity (Figure 2(A)). To ascertain whether **4j** inhibited other AKT isoforms, biochemical IC_50_ analyses were conducted on both purified AKT1/PKBα and AKT2. **4j** exhibited IC_50_s of 12 μM and 14 μM, respectively, AKT2 (Figure 2(B)) and AKT1 (Figure 2(C)).

#### 2.2.3. Akt2 is overexpressed in low-grade glioma and primarily expressed in malignant cells

**Figure 3.**
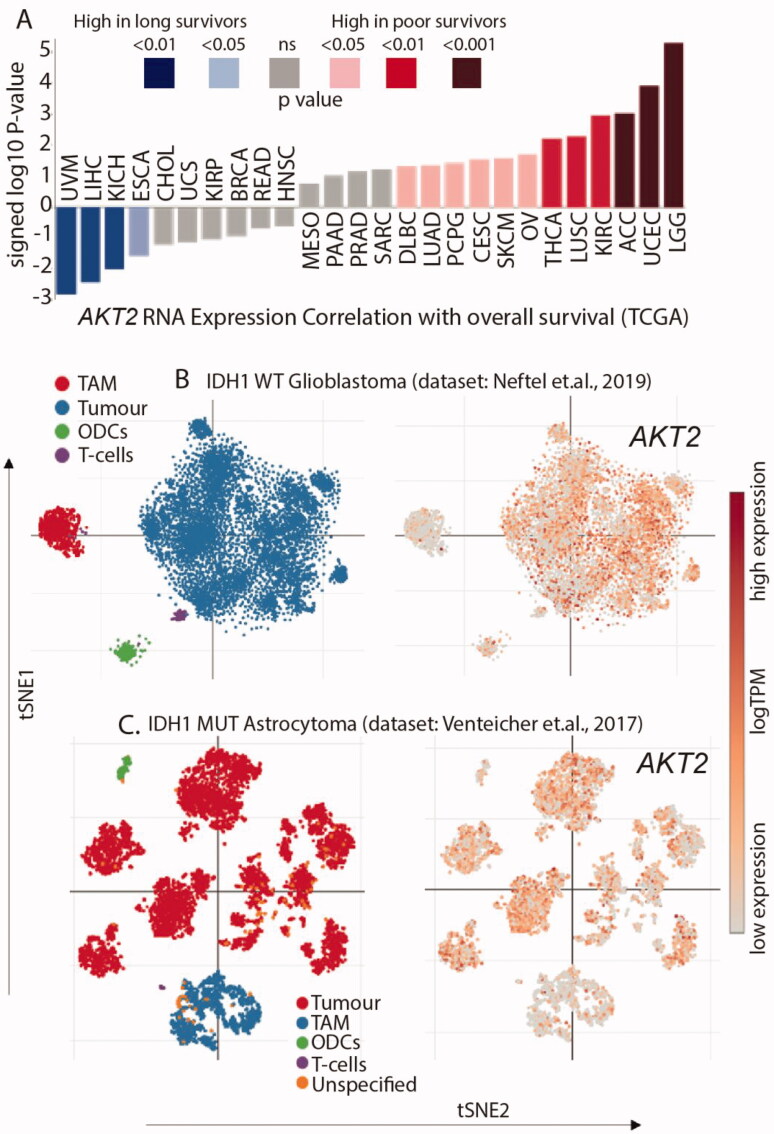
AKT2 is overexpressed in glioma (A) High expression of AKT2 mRNA correlates with poorer survival in LGG patients. Individual values are sign-corrected log10 *p*-values of correlation. Non-abbreviated names of the cancer types: UVM: Uveal Melanoma; LIHC: Liver hepatocellular carcinoma; KICH: Kidney Chromophobe; ESCA: Oesophageal carcinoma; CHOL: Cholangiocarcinoma; UCS: Uterine Carcinosarcoma; KIRP: Kidney renal papillary cell carcinoma; BRCA: Breast invasive carcinoma; READ: Rectum adenocarcinoma; HNSC: Head and Neck squamous cell carcinoma; MESO: Mesothelioma; PAAD: Pancreatic adenocarcinoma; PRAD: Prostate adenocarcinoma; SARC: Sarcoma; DLBC: Lymphoid Neoplasm Diffuse Large B-cell Lymphoma; LUAD: Lung adenocarcinoma; PCPG: Pheochromocytoma and Paraganglioma; CESC: Cervical squamous cell carcinoma and endocervical adenocarcinoma; SKCM: Skin Cutaneous Melanoma; OV: Ovarian serous cystadenocarcinoma; THCA: Thyroid carcinoma; LUSC: Lung squamous cell carcinoma; KIRC: Kidney renal clear cell carcinoma; ACC: Adrenocortical carcinoma; UCEC: Uterine Corpus Endometrial Carcinoma; LGG: Brain Lower Grade Glioma. (B) AKT2 expression is highest in the tumour cell population of IDH1 WT GBM single-cell RNAseq dataset. (C) AKT2 expression is highest in the tumour cell population of IDH1 mutant astrocytoma single-cell RNAseq dataset.

The mRNA expression of AKT2 was correlated with overall survival, which showed that patients with low-grade glioma (LGG) have the highest AKT2 mRNA expression along with the lowest overall survival (Figure 3(A)). To understand the expression levels of AKT2 within the glioma microenvironment and cell states, we queried two widely used single-cell RNA sequencing (scRNAseq) datasets. In the scRNAseq dataset for adult and paediatric IDH1 wild-type glioblastoma, AKT2 expression was maximally identified in the malignant cell population with minimal expression observed in the tumour associated macrophages (TAM), oligodendrocytes (ODCs), and T-cells (Figure 3(B)). A similar expression profile of AKT2 was also observed in the scRNAseq dataset of IDH1 mutant astrocytoma (Figure 3(C)). This suggests that AKT2 is a potential driver of gliomagenesis since the highest expression is observed in malignant cells. This further suggests that targeting AKT2 is a good therapeutic strategy for gliomas.

#### 2.2.4. Establishing cytotoxicity of **4j**

**Figure 4.**
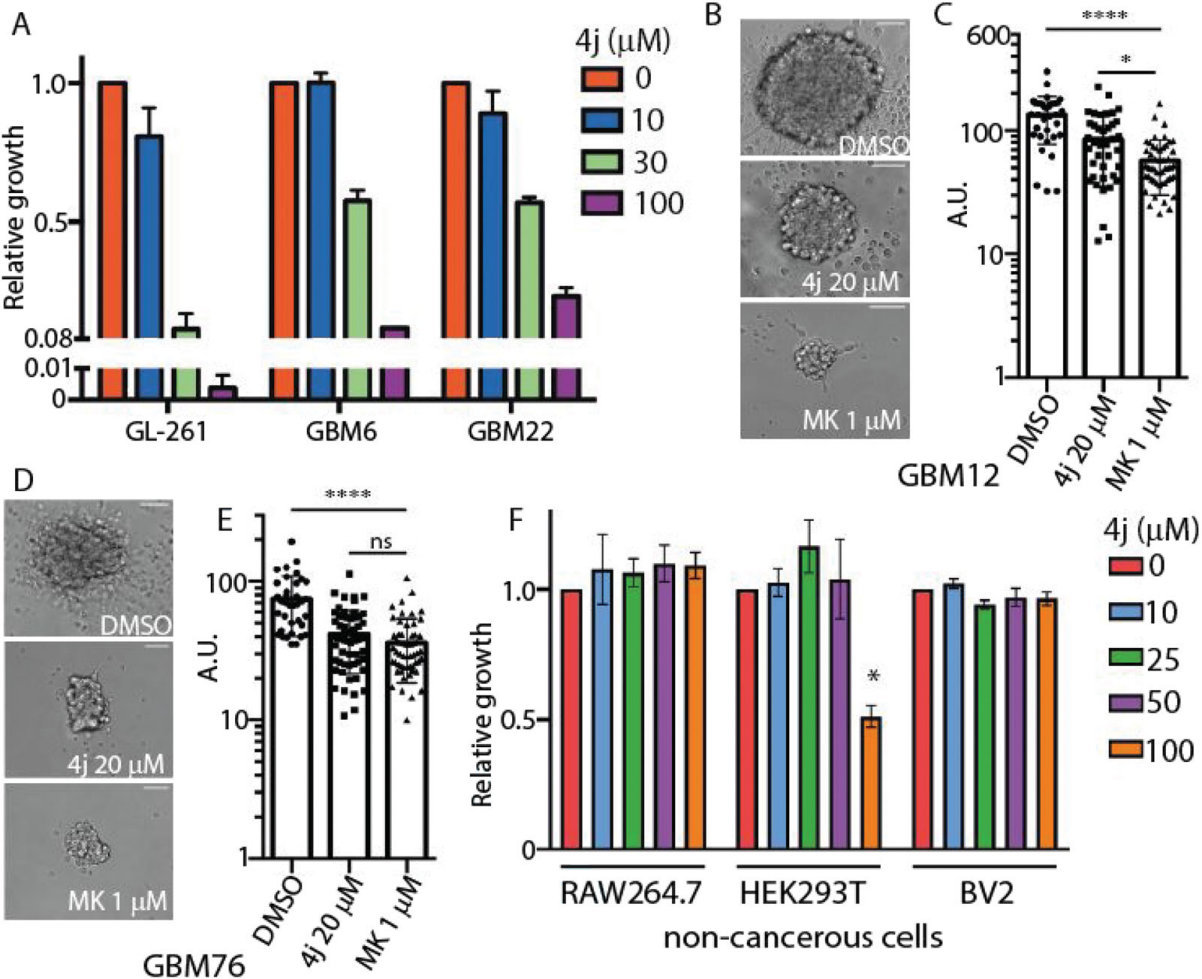
Compound **4j** induces anti-glioma activity in 2D and 3D glioblastoma cell cultures. (A) Glioblastoma cells were treated with the indicated concentrations of **4j** for 72 h, and fold viability was measured using MTS assay. (B) GBM12 cancer stem cells were treated with either DMSO or 20 μM **4j** or 1 μM MK-2206 for 21 days and neurospheres were allowed to form. A representative image of the neurospheres is shown. Scale bar= 125 μm. (C) The diameter of the neurospheres were quantified using ImageJ. The significance of the differences was measured using one-way ANOVA with Tukey’s multiple comparisons. ****p < .0001; *p < .05 (D) As in (B) but GBM76 cancer stem cells were utilised. (E) The diameter of the neurospheres were quantified using ImageJ. The significance of the differences was measured using one-way ANOVA with Tukey’s multiple comparisons. ****p < .0001; ns: not-significant. Scale bar= 125 μm. (F) Non-cancerous RAW264.7, HEK293T, and BV2 cells were treated with the indicated concentrations of **4j** for 72 h, and fold viability was measured using MTS assay. The significance of the differences was measured using two-way ANOVA with Tukey’s multiple comparisons. * = significant.

In order to evaluate the biological activity of **4j**, we utilised immortalised murine glioblastoma cell line GL261 along with primary patient-derived xenograft cells (GBM6, GBM22) and glioblastoma stem 3D neurospheres (GBM12, GBM76). The cell lines and accompanying de-identified histopathological information were established at the Mayo Clinic Brain Tumour PDX National Resource, USA. All three patient-derived primary glioma samples were IDH1 wild-type with *in vivo* bilateral invasive structures observed *via* neuropathology. The effect of **4j** on cell viability was measured against GL261, GBM6, and GBM22 cell lines. After 72 h incubation, cell viability was measured, and the EC_50_ values for **4j** in GL261 are approximately 20 μM and 30 μM in GBM6 and GBM22 (Figure 4(A)). To further determine whether **4j** treatment could impact a 3 D glioma stem cell culture system, we treated GBM12 and GBM76 glioma stem cell lines with 20 μM **4j** or 1 µM established PKB inhibitor MK-2206 for 21 days. **4j** treated GBM12 neurospheres were significantly smaller in size compared to DMSO control as observed under bright-field (Figure 4(B,C)). MK-2206 treated GBM12 neurospheres were significantly smaller than **4j** treated (Figure 4(C)). However, in the case of GBM76, no difference in diameter size was observed between 1 µM MK-2206-treated and 20 µM **4j**-treated 3D neurospheres (Figure 4(D,E)). Both **4j** and MK-2206 equally reduced GBM76 neurosphere sizes. Interestingly, **4j** did not induce any cytotoxicity in non-cancerous myeloid cells RAW264.7 (human) or BV2 (murine) with modest but statistically significant cell death observed in human embryonic kidney HEK293T cells at high 100 μM dose (Figure 4(F)). This suggests **4j** is specific for glioma cells and potentially nontoxic.

## 3. Conclusion

A series of *N*-(4-chlorophenyl) substituted pyrano[2,3-c]pyrazole derivatives was synthesised (Figure 1 & Scheme 3) and compound **4j** was determined to be a kinase inhibitor with anti-glioma activity. Upon kinase screening and IC_50_ analyses, compound **4j** showed inhibitory activity towards AKT2/PKBβ (Figure 2). Aberrant AKT2 signalling is common in many cancers, such as glioma. We queried the cancer genome atlas database and utilised bioinformatic analysis to report that AKT2 mRNA was highest in low-grade glioma, and also the high expression correlates with poor patient survival (Figure 3(A)). Furthermore, *in silico* analysis of scRNA-seq data of glioma tumours, we found that AKT2 expression is highest in the malignant tumour cells themselves when compared to the immune and stromal populations (Figure 3(B,C)). Upon treating murine and patient-derived glioma cell lines with **4j**, EC_50_ values were 18–30 μM (Figure 4(A)). Treating patient derived glioma stem 3D neurospheres with **4j** also significantly reduced neurosphere size (Figure 4(B–E)), this suggesting potent anti-glioma activity in stem-like organoid models. Established, highly potent, and clinically relevant AKT inhibitor MK-2206 exhibits ∼20-fold more potency in targeting 2D glioma cells (Table 1) and 3D glioma neurospheres than **4j**. However, it is important to note that the MK-2206 biochemical IC_50_ for AKT2 is 12 nM [40], which is 1000-fold higher than 12 μM of **4j**, MK-2206 was expected to exhibit very high potency in glioma where AKT expression and signalling pathway are amplified [10]. Furthermore, the most important aspect of **4j** is that it is nontoxic to non-cancerous myeloid and kidney cells (Figure 4(F)) which suggests that potential toxic side effects could be reduced if the pyranopyrazoles were explored with further medicinal chemistry. HEK293 lineage of cells are non-cancerous, however, they do exhibit enhanced AKT1/PKBα signalling which could explain its marginal sensitivity to **4j** at 100 μM [41]. It is also possible that **4j** has other non-kinase targets in the glioma cell, and we are cognisant of the fact that further optimisations and evaluations are required to establish clinical relevance for pyrano[2,3-c]pyrazoles. Currently the pyranopyrazoles are at an early stage of development and through this study we have identified a lead compound **4j**. The molecule lacks predicted blood-brain barrier penetrance, kinetic solubility, and exhibits high topological polar surface area. Hence, further medicinal chemistry is required for lead optimisation and eventual pre-clinical evaluation *in vivo* in glioma xenograft models. We are currently engaged in extensive structure–activity relationship driven drug development programme to develop pyranopyrazoles as viable anticancer drugs. Gliomas being one of the most common brain cancers, there is always a need for more therapeutic strategies and our novel nontoxic molecule is the ideal starting point for the medicinal chemistry programme. Here, we have established a novel pyrano[2,3-c]pyrazole compound **4j,** enabling kinase inhibitory and anti-glioma activity for the first time.

## 4. Experimental section

### 4.1. Chemistry

#### 4.1.1. General

We purchased 1,4 Diazabicyclo[2.2.2]octane (DABCO) from Alfa Aesar Chemical Company. 4-Ethoxy benzaldehyde, 4-phenyl benzaldehyde and 1-(4-chlorophenyl)-3-methyl-5-pyrazalone were purchased from TCI Chemicals (India) Pvt. Ltd. Benzaldehyde, p-tolualdehyde, 4-nitro benzaldehyde, 4-chloro benzaldehyde, and 4-hydroxy benzaldehyde were purchased from Sisco Research Laboratories Pvt. Ltd. 4-Bromo benzaldehyde and 4-fluoro benzaldehyde were purchased from Spectrochem Pvt. Ltd. ^1^H and ^13^C{^1^H} NMR spectra were recorded on a Bruker AV-400 or Bruker AV-600 device. Chemical shifts are expressed in ppm relative to TMS in DMSO-d6. High-resolution mass spectra were obtained on a Bruker Compass Data Analysis 4.0 spectrometer. Pyrano[2,3-c]pyrazoles with labelled atoms are shown in Scheme 3. For NMR spectra and HRMS details, please refer to Supporting Information Figures S2–S32.

#### 4.1.2. General procedure for the synthesis of pyrano[2,3-c]pyrazoles

In a round bottom flask, 1 mmol aldehyde, 1 mmol 1-(4-chlorophenyl)-3-methyl-5-pyrazolone, 1.1 mmol malononitrile and 0.1 mmol DABCO were mixed in 5 mL ethanol. The reaction mixture was refluxed and stirred at 78 °C for 30 min in a water bath. The water bath temperature should be below 80 °C; otherwise, it caused the reaction mixture to become more brownish, which increases effort in the workup process. In case the reaction mixture solidified, extra 3 mL ethanol was added. The progress of the reaction was monitored by TLC (hexane/ethyl acetate = 1/1). After completion, the reaction mixture was cooled to room temperature and filtered to separate the product. The product was washed with warm water three times to remove colour impurity.

##### 4.1.2.1. 6-Amino-1-(4-chlorophenyl)-3-methyl-4-phenyl-1,4-ihydropyrano[2,3-c]pyrazole-5-carbonitrile (4a)

White solid (0.277 g, 77%), mp: 196–198 °C; ^1^H NMR (400 MHz, DMSO-d6) δ: 1.78 (s, 3H, CH_3_), 4.68 (s, 1H, CH), 7.26–7.28 (m, 5H, NH_2_, Ar-H), 7.35(t, *J* = 7.4 Hz, 2H, Ar-H), 7.54 (d, *J* = 8.8 Hz, 2H, Ar-H), 7.83 (d, *J* = 8.8 Hz, 2H, Ar-H); ^13^C{^1^H} NMR (100 MHz, DMSO-d6) δ: 12.6 (CH_3_), 36.7 (CH), 58.2 (C-5), 98.9(C-3a), 119.9 (CN), 121.4 (Ar-C), 127.1 (Ar-C), 127.8 (Ar-C), 128.5 (Ar-C), 129.2 (Ar-C), 130.2 (Ar-C), 136.4 (Ar-C), 143.5 (Ar-C), 144.0 (C-3), 145.7 (C-7a), 159.3 (C-6); HRMS (ESI-TOF) *m/z* calcd. for C_20_H_15_ClN_4_O (M + H)^+^: 363.10126, found: 363.10024.

##### 4.1.2.2. 6-Amino-1-(4-chlorophenyl)-3-methyl-4-p-tolyl-1,4-dihydropyrano[2,3-c]pyrazole-5-carbonitrile (4b)

White solid (0.298 g, 79%), mp: 210–212 °C; ^1^H NMR (400 MHz, DMSO-d6) δ: 1.78 (s, 3H, CH_3_), 2.29 (s, 3H, CH_3_), 4.62 (s, 1H, CH), 7.14 (s, 4H, Ar-H), 7.23 (s, 2H, NH_2_), 7.53 (d, *J* = 8.8 Hz, 2H, Ar-H), 7.83 (d, *J* = 8.8 Hz, 2H, Ar-H); ^13^C{^1^H} NMR (100 MHz, DMSO-d6) δ: 12.6 (CH_3_), 20.7(CH_3_), 36.3 (CH), 58.4 (C-5), 99.0(C-3a), 119.9 (CN), 121.3 (Ar-C), 127.7 (Ar-C), 129.1 (Ar-C), 129.2 (Ar-C), 130.1 (Ar-C), 136.1 (Ar-C), 136.4 (Ar-C), 140.5 (Ar-C), 143.9(C-3), 145.8 (C-7a), 159.2(C-6); HRMS (ESI-TOF) *m/z* calcd. for C_21_H_17_ClN_4_O (M + H)^+^: 377.11691, found: 377.11585.

##### 4.1.2.3. 6-Amino-1-(4-chlorophenyl)-3-methyl-4-(4-nitrophenyl)-1,4-dihydropyrano[2,3-c]pyrazole-5-carbonitrile (4c)

Light yellow solid (0.341 g, 84%), mp: 228–230 °C; ^1^H NMR (400 MHz, DMSO-d6) δ: 1.79 (s, 3H, CH_3_), 4.93 (s, 1H, CH), 7.42 (s, 2H, NH_2_), 7.57 (dd, *J* = 8.8 Hz, 17.6 Hz, 4H, Ar-H), 7.84 (d, *J* = 8.8 Hz, 2H, Ar-H), 8.23 (d, *J* = 8.8 Hz, 2H, Ar-H); ^13^C{^1^H} NMR (100 MHz, DMSO-d6) δ: 12.6 (CH_3_), 36.3 (CH), 56.9 (C-5), 97.8(C-3a), 119.7 (CN), 121.5 (Ar-C), 123.9 (Ar-C), 129.2 (Ar-C), 130.4 (Ar-C), 136.3 (Ar-C), 144.1(C-3), 145.6 (C-7a), 146.6 (Ar-C), 151.1 (Ar-C), 159.6(C-6); HRMS (ESI-TOF) *m/z* calcd. for C_20_H_14_ClN_5_O_3_ (M + H)^+^: 408.08634, found: 408.08520.

##### 4.1.2.4. 6-Amino-1,4-bis(4-chlorophenyl)-3-methyl-1,4-dihydropyrano[2,3-c]pyrazole-5-carbonitrile (4d)

White solid (0.299, 75%), mp: 218–220 °C; ^1^H NMR (400 MHz, DMSO-d6) δ: 1.79 (s, 3H, CH_3_), 4.73 (s, 1H, CH), 7.30–7.32 (m, 4H, NH_2_, Ar-H), 7.41 (d, *J* = 8.4 Hz, 2H, Ar-H), 7.54 (d, *J* = 8.8 Hz, 2H, Ar-H), 7.83 (d, *J* = 8.8 Hz, 2H, Ar-H); ^13^C{^1^H} NMR (100 MHz, DMSO-d6) δ: 12.6 (CH_3_), 36.0 (CH), 57.7 (C-5), 98.4(C-3a), 119.8 (CN), 121.4 (Ar-C), 128.5 (Ar-C), 129.2 (Ar-C), 129.7 (Ar-C), 130.3 (Ar-C), 131.6 (Ar-C), 136.3 (Ar-C), 142.6 (Ar-C), 144.0 (C-3), 145.7 (C-7a), 159.4(C-6); HRMS (ESI-TOF) *m/z* calcd. for C_20_H_14_Cl_2_N_4_O (M + H)^+^: 397.06229, found: 397.06122.

##### 4.1.2.5. 6-Amino-4-(4-bromophenyl)-1-(4-chlorophenyl)-3-methyl-1,4-dihydropyrano[2,3-c]pyrazole-5-carbonitrile (4e)

White solid (0.344 g, 78%), mp: 224–226 °C; ^1^H NMR (400 MHz, DMSO-d6) δ: 1.79 (s, 3H, CH_3_), 4.71 (s, 1H, CH), 7.25 (d, *J* = 8.4 Hz, 2H, Ar-H), 7.31 (s, 2H, NH_2_), 7.52–7.56 (m, 4H, Ar-H), 7.83 (d, *J* = 8.8 Hz, 2H, Ar-H); ^13^C{^1^H} NMR (100 MHz, DMSO-d6) δ: 12.6 (CH_3_), 36.1 (CH), 57.6 (C-5), 98.4(C-3a), 119.8 (CN), 120.2 (Ar-C), 121.4 (Ar-C), 129.2 (Ar-C), 130.1 (Ar-C), 130.3 (Ar-C), 131.5 (Ar-C), 136.3 (Ar-C), 143.0 (Ar-C), 144.0 (C-3), 145.7 (C-7a), 159.4(C-6); HRMS (ESI-TOF) m/z calcd. for C_20_H_14_BrClN_4_O (M + H)^+^: 441.01178, found: 441.01042.

##### 4.1.2.6. 6-Amino-1-(4-chlorophenyl)-4-(4-fluorophenyl)-3-methyl-1,4-dihydropyrano[2,3-c]pyrazole-5-carbonitrile (4f)

White solid (0.305 g, 80%), mp: 192–194 °C; ^1^H NMR (400 MHz, DMSO-d6) δ: 1.78 (s, 3H, CH_3_), 4.72 (s, 1H, CH), 7.17 (t, *J* = 8.4 Hz, 2H, Ar-H), 7.28 (s, 2H, NH_2_) 7.30–7.33 (m, 2H, Ar-H), 7.54 (d, *J* = 8.8 Hz, 2H, Ar-H), 7.83 (d, *J* = 8.8 Hz, 2H, Ar-H); ^13^C{^1^H} NMR (100 MHz, DMSO-d6) δ: 12.6 (CH_3_), 35.9 (CH), 58.0 (C-5), 98.7(C-3a), 115.3 (d, *J* = 22 Hz, Ar-C),119.9 (CN), 121.4 (Ar-C), 129.2 (Ar-C), 129.7 (d, *J* = 8 Hz, Ar-C), 130.2 (Ar-C), 136.4 (Ar-C), 139.7 (d, *J* = 3 Hz, Ar-C), 144.0 (C-3), 145.7(C-7a), 159.3(C-6), 161.2 (d, *J* = 241 Hz, Ar-C); HRMS (ESI-TOF) *m/z* calcd. for C_20_H_14_ClFN_4_O (M + H)^+^: 381.09184, found: 381.09077.

##### 4.1.2.7. 6-Amino-1-(4-chlorophenyl)-4-(4-hydroxyphenyl)-3-methyl-1,4-dihydropyrano[2,3-c]pyrazole-5-carbonitrile (4g)

White solid (0.310 g. 82%), mp: 212–214 °C; ^1^H NMR (400 MHz, DMSO-d6) δ: 1.79 (s, 3H, CH_3_), 4.56 (s, 1H, CH), 6.72 (d, *J* = 8.4 Hz, 2H, Ar-H), 7.04 (d, *J* = 8.4 Hz, 2H, Ar-H), 7.18 (s, 2H, NH_2_), 7.53 (d, *J* = 8.8 Hz, 2H, Ar-H), 7.82 (d, *J* = 8.8 Hz, 2H, Ar-H), 9.36 (s, 1H, OH); ^13^C{^1^H} NMR (100 MHz, DMSO-d6) δ: 12.6 (CH_3_), 36.0 (CH), 58.8 (C-5), 99.3(C-3a), 115.2 (Ar-C), 120.0 (CN), 121.3 (Ar-C), 128.8 (Ar-C), 129.2 (Ar-C), 130.1 (Ar-C), 133.8 (Ar-C), 136.4 (Ar-C), 143.8 (C-3), 145.8(C-7a), 156.3 (Ar-C), 159.1(C-6); HRMS (ESI-TOF) *m/z* calcd. for C_20_H_15_ClN_4_O_2_ (M + H)^+^: 379.09618, found: 379.09505.

##### 4.1.2.8. 6-Amino-1-(4-chlorophenyl)-4-(4-methoxyphenyl)-3-methyl-1,4-dihydropyrano[2,3-c]pyrazole-5-carbonitrile (4h)

White solid (0.343 g, 87%), mp: 218–220 °C; ^1^H NMR (400 MHz, DMSO-d6) δ: 1.78 (s, 3H, CH_3_), 3.75 (s, 3H, CH_3_), 4.63 (s, 1H, CH), 6.90 (d, *J* = 8.4 Hz, 2H, Ar-H), 7.17 (d, *J* = 8.4 Hz, 2H, Ar-H), 7.21 (s, 2H, NH_2_), 7.53 (d, *J* = 8.8 Hz, 2H, Ar-H), 7.83 (d, *J* = 8.8 Hz, 2H, Ar-H); ^13^C{^1^H} NMR (100 MHz, DMSO-d6) δ: 12.6 (CH_3_), 35.9 (CH), 55.0(OCH_3_), 58.5 (C-5), 99.1(C-3a), 113.8 (Ar-C), 120.0(CN), 121.3 (Ar-C), 128.9 (Ar-C), 129.2 (Ar-C), 130.1 (Ar-C), 135.5 (Ar-C), 136.4 (Ar-C), 143.9 (C-3), 145.8(C-7a), 158.2 (Ar-C), 159.2(C-6); HRMS (ESI-TOF) *m/z* calcd. for C_21_H_17_ClN_4_O_2_ (M + H)^+^: 393.111829, found: 393.11077.

##### 4.1.2.9. 6-Amino-1-(4-chlorophenyl)-4-(4-ethoxyphenyl)-3-methyl-1,4-dihydropyrano[2,3-c]pyrazole-5-carbonitrile (4i)

White solid (0.322 g, 79%), mp: 190–192 °C; ^1^H NMR (400 MHz, DMSO-d6) δ: 1.32 (t, *J* = 6.8 Hz, 3H, CH_3_), 1.78 (s, 3H, CH_3_), 4.00 (q, *J* = 6.8 Hz, 2H, CH_2_), 4.62 (s, 1H, CH), 6.88 (d, *J* = 8.4 Hz, 2H, Ar-H), 7.15 (d, *J* = 8.8 Hz, 2H, Ar-H), 7.21 (s, 2H, NH_2_), 7.53 (d, *J* = 8.8 Hz, 2H, Ar-H), 7.83 (d, *J* = 8.8 Hz, 2H, Ar-H); ^13^C{^1^H} NMR (100 MHz, DMSO-d6) δ: 12.6 (CH_3_), 14.7 (CH_3_), 35.9 (CH), 58.6 (C-5), 62.9(OCH_2_), 99.1(C-3a), 114.3 (Ar-C), 120.0(CN), 121.3 (Ar-C), 128.8 (Ar-C), 129.2 (Ar-C), 130.1 (Ar-C), 135.4 (Ar-C), 136.4 (Ar-C), 143.9 (C-3), 145.8(C-7a), 157.5 (Ar-C), 159.2(C-6); HRMS (ESI-TOF) *m/z* calcd. for C_22_H_19_ClN_4_O_2_ (M + H)^+^: 407.12748, found: 407.12642.

##### 4.1.2.10. 6-Amino-4-(biphenyl-4-yl)-1-(4-chlorophenyl)-3-methyl-1,4-dihydropyrano[2,3-c]pyrazole-5-carbonitrile (4j)

White solid (0.390 g, 89%), mp: 204–206 °C; ^1^H NMR (400 MHz, DMSO-d6) δ: 1.84 (s, 3H, CH_3_), 4.75 (s, 1H, CH), 7.29 (s, 2H, NH_2_), 7.35–7.38 (m, 3H, Ar-H), 7.47 (t, *J* = 7.6 Hz, 2H), 7.55 (d, *J* = 8.8 Hz, 2H, Ar-H), 7.69 (t, *J* = 7.4 Hz, 4H, Ar-H), 7.85 (d, *J* = 8.8 Hz, 2H, Ar-H); ^13^C{^1^H} NMR (100 MHz, DMSO-d6) δ: 12.6 (CH_3_), 36.3 (CH), 58.0 (C-5), 98.8 (C-3a), 120.0(CN), 121.4 (Ar-C), 126.6 (Ar-C), 126.9 (Ar-C), 127.4 (Ar-C), 128.4 (Ar-C), 128.9 (Ar-C), 129.2 (Ar-C), 130.2 (Ar-C), 136.4 (Ar-C), 138.9 (Ar-C), 139.7 (Ar-C), 142.7 (Ar-C), 144.0 (C-3), 145.8 (C-7a), 159.4 (C-6); HRMS (ESI-TOF) *m/z* calcd. for C_26_H_19_ClN_4_O (M + H)^+^: 439.13256, found: 439.13138.

### 4.2. Biochemical analysis

#### 4.2.1. Kinase screen analysis of 4j

Kinase inhibitor specificity profiling assays were carried out at The International Centre for Protein Kinase Profiling (http://www.kinase-screen.mrc.ac.uk/). **4j** biochemical kinase inhibitory property was determined against a panel of 139 protein kinases as described previously [42,43]. The assay mixes and ^33^P-γ-ATP were added by Multidrop 384 (Thermo). Results are presented as a percentage of kinase activity in DMSO control reactions. Protein kinases were assayed *in vitro* with 5 μM final concentration of **4j,** and the results are presented as an average of triplicate reactions in the form of comparative histograms using Adobe Illustrator.

#### 4.2.2. IC_50_ analyses

IC_50_ analyses were carried out at The International Centre for Protein Kinase Profiling (http://www.kinase-screen.mrc.ac.uk/). **4j** IC_50_ measurements were carried out against the kinases with final concentrations between 0.003 to 100 μM *in vitro* (**4j** was added to the kinase reaction prior to ATP master mix). PKBβ (ΔPH-PKBβ-S474D) and PKBα (ΔPH-PKBα-S473D) were purified using a baculoviral expression system as stated previously [42]. PKBβ or PKBα (5–20 mU diluted in 50 mM Tris pH 7.5, 0.1 mM EGTA, 0.1% β-mercaptoethanol, 1 mg/mL bovine serum albumin) were assayed against a modified Crosstide peptide (GRPRTSSFAEGKK) in a final volume of 25.5 µL containing 50 mM Tris pH 7.5, 0.05% β-mercaptoethanol, 30 µM substrate peptide, 10 mM magnesium acetate and 0.05 mM [^33^P-γ-ATP] (50–1000 cpm/pmole) and incubated for 30 min at room temperature. Assays were stopped by adding 5 µL of 0.5 M (3%) orthophosphoric acid and then harvested onto P81 Unifilter plates with a wash buffer of 50 mM orthophosphoric acid. IC_50_ curves were developed as % of DMSO control, and IC_50_ values were calculated using GraphPad Prism software.

#### 4.2.3. Bioinformatic analysis

##### 4.2.3.1. Expression correlation with overall survival for tumour indication in TCGA

The AKT2 expression correlation with overall survival for tumour indication is based upon data generated through the Lumin Bioinformatics Software of Champions Oncology, Inc. This signature reflects the correlation of AKT2 mRNA expression with the overall survival in each of the TCGA cancer indications described. The individual values are sign-corrected log10 *p*-values of correlation. For example, a value of −2, (representing a *p*-value of .01), for AKT2 indicates that when AKT2 expression is high, patient outcome is better with longer survival, whereas a positive 2 value (representing a *p*-value of .01), indicates the opposite: high expression of AKT2 predicts worse outcome or poorer survival.

##### 4.2.3.2. AKT2 expression analysis from single-cell RNA sequencing datasets

To understand expression across various cell states of glioblastoma, we queried AKT2 levels in two previously published single-cell RNA sequencing datasets available on the Single Cell portal of Broad Institute, MIT and Harvard, USA (https://singlecell.broadinstitute.org/single_cell). We explored the “Single-cell RNA-seq of adult and paediatric glioblastoma” dataset consisting of 24,131 single-cell sequences from patients with IDH1 wild-type glioblastoma [44] and the “single-cell RNA-seq analysis of astrocytoma” dataset consisting of 6341 single-cell sequences from 10 patients with IDH1 mutant astrocytoma [45]. Data represented with t-distributed stochastic neighbour embedding (t-SNE) clustering and sub-sampling of “All Cells.”

### 4.3. Cell-based assays

#### 4.3.1. Cell culture

Mammalian cells were all grown in a humidified incubator with 5% CO_2_ at 37 °C. GL261 (gift from Prof Kun-Liang Guan, University of California San Diego, USA), RAW264.7, HEK293T, and BV2 (all 3 cell lines purchased from ATCC) cells were cultured in Dulbecco’s Modified Eagle Media (DMEM, Gibco) supplemented with 10% FBS and 1% penicillin and streptomycin. GBM6 and GBM22 cells were cultured in DMEM supplemented with 10% FBS, 1% penicillin and streptomycin, 10 μg/mL insulin, and 20 ng/mL hEGF. GBM12 and GBM76 cells were cultured in neurosphere media consisting of KnockOut DMEM/F-12 Basal Media supplemented with StemPro NSC SFM Supplement, 10 µg FGF, 10 µg EGF, L-glutamine (Corning #25005CI) 10 mL of 200 mM solution, and 1% penicillin and streptomycin. GBM6, GBM12, GBM22, and GBM76 cells were acquired from the Brain Tumour PDX National Resource, Mayo Clinic, USA. Resected tumours from patients were propagated subcutaneously in immunocompromised mice and the tumours were dissociated and cultured in either growth media with FBS or neurosphere media in stem conditions. Immunophenotyping has not been carried out on these tumours. All primary GBM cells were established by the Mayo Clinic (https://www.mayo.edu/research/labs/translational-neuro-oncology/mayo-clinic-brain-tumour-patient-derived-xenograft-national-resource/protocols).

#### 4.3.2. Cell viability assay

Cell viability assays were carried out as stated previously [46–48]. To measure cell viability, actively proliferating cells were seeded at with an equal number of cells per well. Cell viability assays were carried out with or without 72 h treatment of the respective inhibitor using the CellTiter 96® AQueous Non-Radioactive Cell Proliferation Assay kit following manufacturer’s instructions, and data was represented as relative viability compared to DMSO treated control.

#### 4.3.3. Neurosphere formation assay

A neurosphere formation assay is a cellular event wherein the 2D primary glioma cells aggregate to form a 3D organoid structure (also referred to as a neurosphere) when cultured in stem cell media. GBM12 and GBM76 cells were plated at 4000 cells per well in neurosphere media supplemented with either DMSO, 20 μM **4j** or 1 μM MK-2206 (Selleckchem #S1078) for 21 days in triplicates. After 21 days, representative images were taken of each well using the Zeiss Axiovert Live microscope. Diameters of the neurospheres were quantified using ImageJ software, and graphs were plotted on GraphPad Prism.

### 4.4. Statistical analysis

Details of all statistical tests and multiple comparisons used to derive *p*-value has been detailed in figure legends. All experiments were repeated 2–3 times with multiple technical replicates to be eligible for the indicated statistical analyses, and representative images were shown. All results are presented as mean ± *SD* unless otherwise mentioned. Data were analysed using GraphPad Prism statistical package unless otherwise mentioned. TCGA data mining and corresponding statistical analyses were performed by Lumin Bioinformatics webtool, as stated previously.

## Supplementary Material

Supplemental Material

## Data Availability

All data that support these findings of this study are included in this manuscript. Further information and chemical reagents are available upon request to the co-corresponding author HMP. Queries pertaining to the biological evaluations should be directed to the co-corresponding author SB.

## Abbreviations

ATCC: American Type Culture Collection
DABCO: 1,4-diazabicyclo[2.2.2]octane
EGF: epidermal growth factor
FGF: fibroblast growth factor
GBM: glioblastoma multiforme
HRMS: High-resolution mass spectrometry
IDH: isocitrate dehydrogenase
NMR: nuclear magnetic resonance
PKB: Protein Kinase B
TCGA: The Cancer Genome Atlas
