# Supplementary material for: Synthesis of N-(4-chlorophenyl) substituted pyrano[2,3-c]pyrazoles enabling PKBβ/AKT2 inhibitory and in vitro anti-glioma activity
Source: Ann Med. 2022 Sep 18;54(1):2549–61. doi: 10.1080/07853890.2022.2123559 (PMC9683054; doi:10.1080/07853890.2022.2123559)
Supplement: Supplemental Material [file IANN_A_2123559_SM3714.docx]

**Supporting Information**

Synthesis of *N*-(4-chlorophenyl) substituted pyrano[2,3-c]pyrazoles enabling PKBβ/AKT2 inhibitory and *in vitro* anti-glioma activity

Ruturajsinh M Vala ^a,d^, Vasudha Tandon ^b,d^, Lynden G. Nicely ^b^, Luxia Guo ^c^, Yanlong Gu ^c^, Sourav Banerjee ^b,^*, Hitendra M. Patel ^a,^*

^a^ Department of Chemistry, Sardar Patel University, Vallabh Vidyanagar 388120, Gujarat, India. ^b^ Department of Cellular Medicine, School of Medicine, University of Dundee, Dundee DD1 9SY, UK. ^c^ Key Laboratory of Material Chemistry for Energy Conversion and Storage, Ministry of Education, Hubei, Key Laboratory of Material Chemistry and Service Failure, School of Chemistry and Chemical Engineering, Huazhong University of Science and Technology, 1037 Luoyu Road, Hongshan District, Wuhan 430074, China

^d^ Authors contributed equally

*Corresponding author: Sourav Banerjee E-mail: [s.y.banerjee@dundee.ac.uk](mailto:s.y.banerjee@dundee.ac.uk), Department of Cellular Medicine, School of Medicine, University of Dundee, Dundee DD1 9SY, UK; Hitendra M. Patel Email: [hm_patel@spuvvn.edu](mailto:hm_patel@spuvvn.edu), Department of Chemistry, Sardar Patel University, Vallabh Vidyanagar 388120, Gujarat, India

**Table of Contents**

| 1 | Copies of ^1^H NMR and ^13^C{^1^H} NMR spectra of **4a**-**4j** | S2-S12 |
| --- | --- | --- |
| 2 | Copies of HRMS of **4a**-**4j** | S14-S22 |

1. Copies of ^1^H NMR and ^13^C{^1^H} NMR spectra of **4a**-**4j**


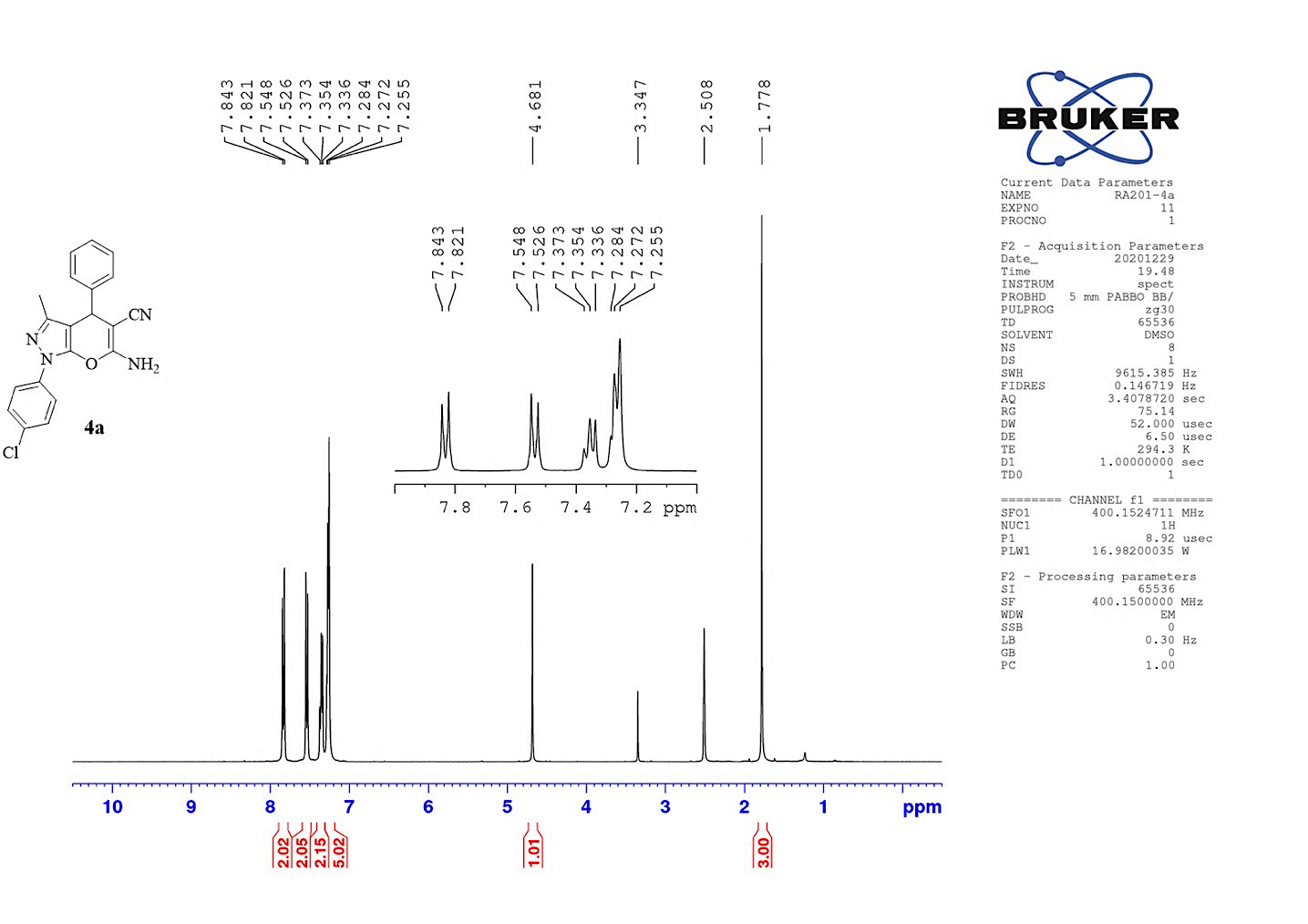


**Figure S1.** ^1^H NMR spectrum of compound **4a** at 400 MHz in DMSO-d_6_

_
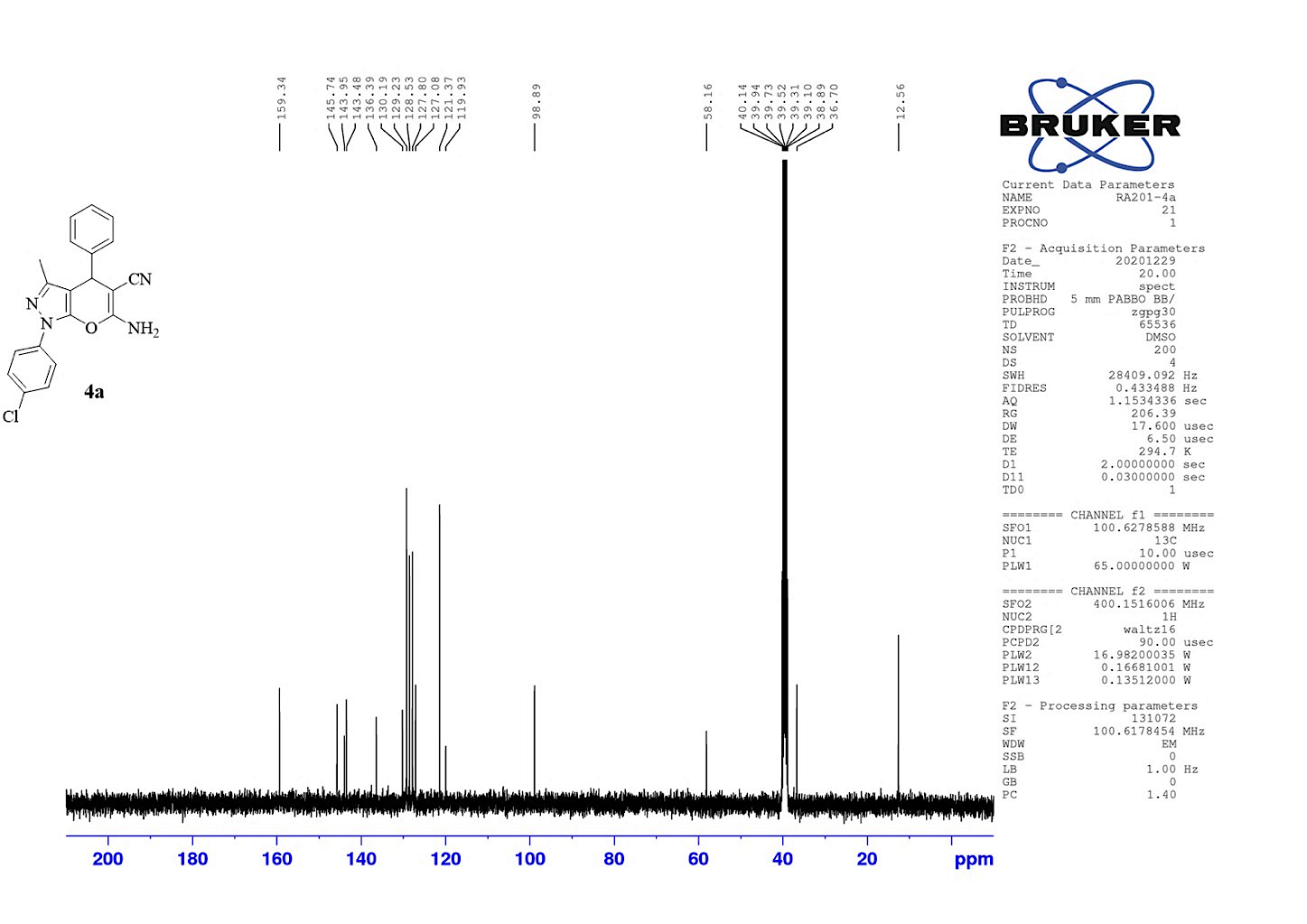
_

**Figure S2.** ^13^C{^1^H} NMR spectrum of compound **4a** at 100 MHz in DMSO-d_6_

_
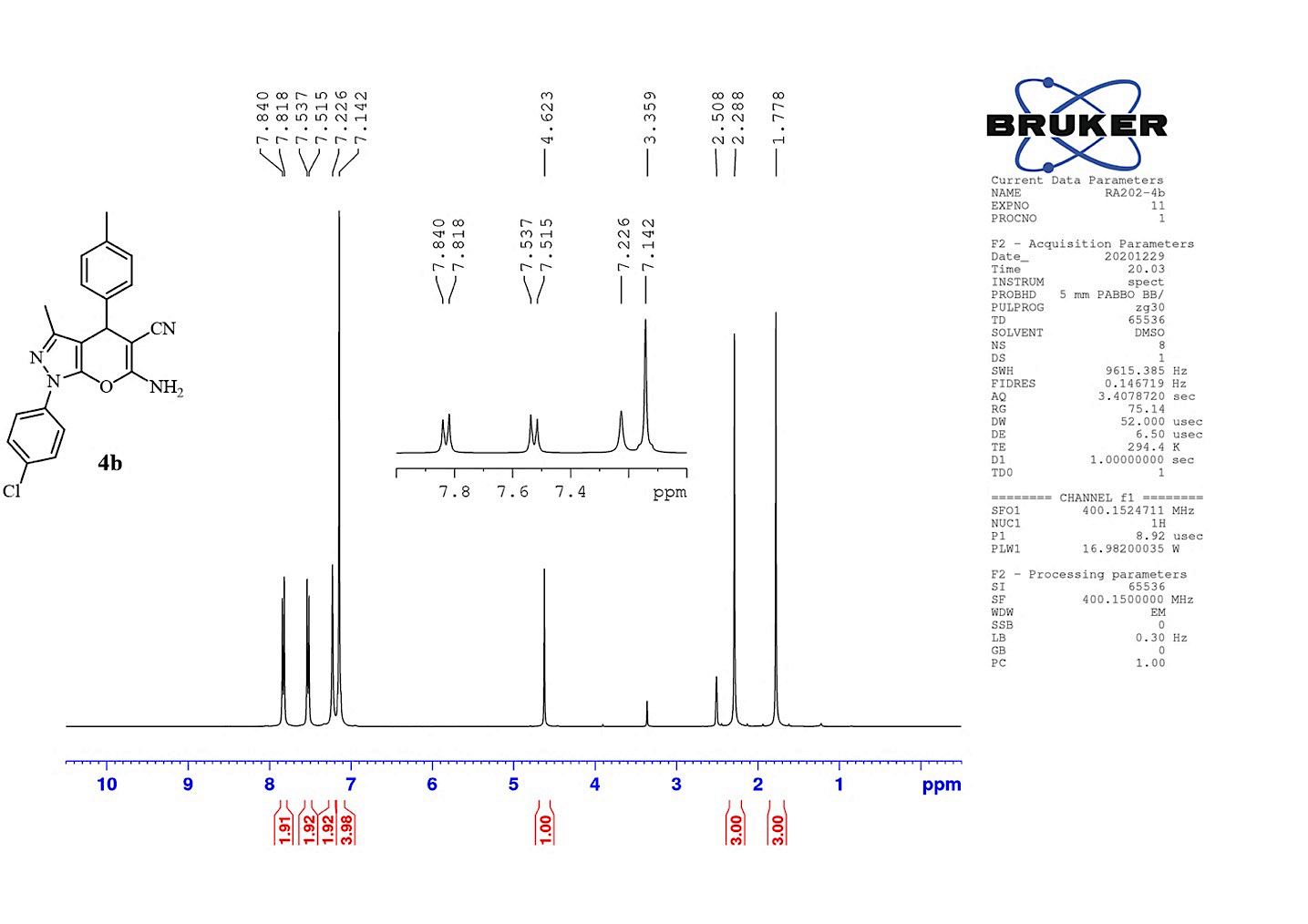
_

**Figure S3.** ^1^H NMR spectrum of compound **4b** at 400 MHz in DMSO-d_6_

_
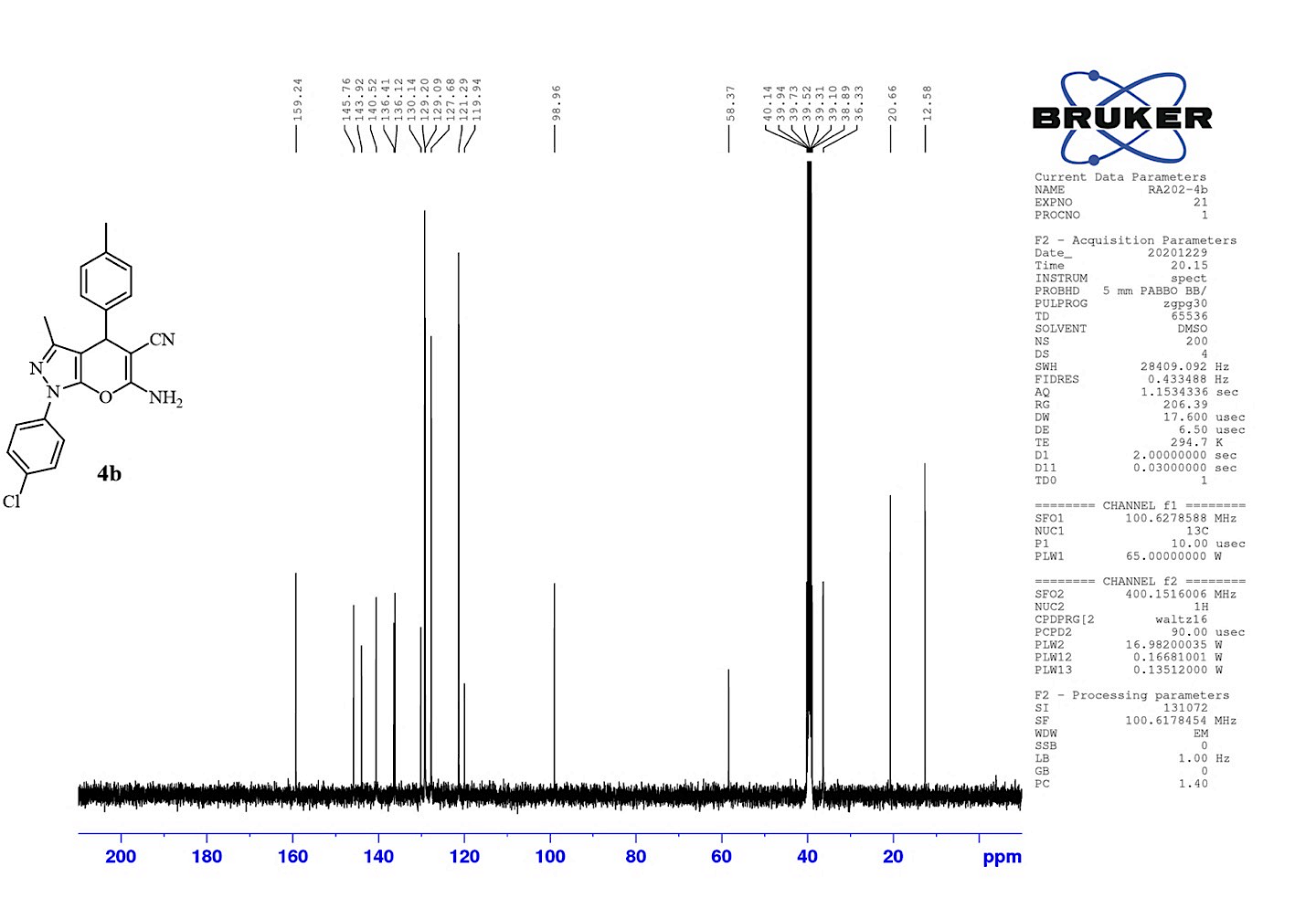
_

**Figure S4.** ^13^C{^1^H} NMR spectrum of compound **4b** at 100 MHz in DMSO-d_6_

**
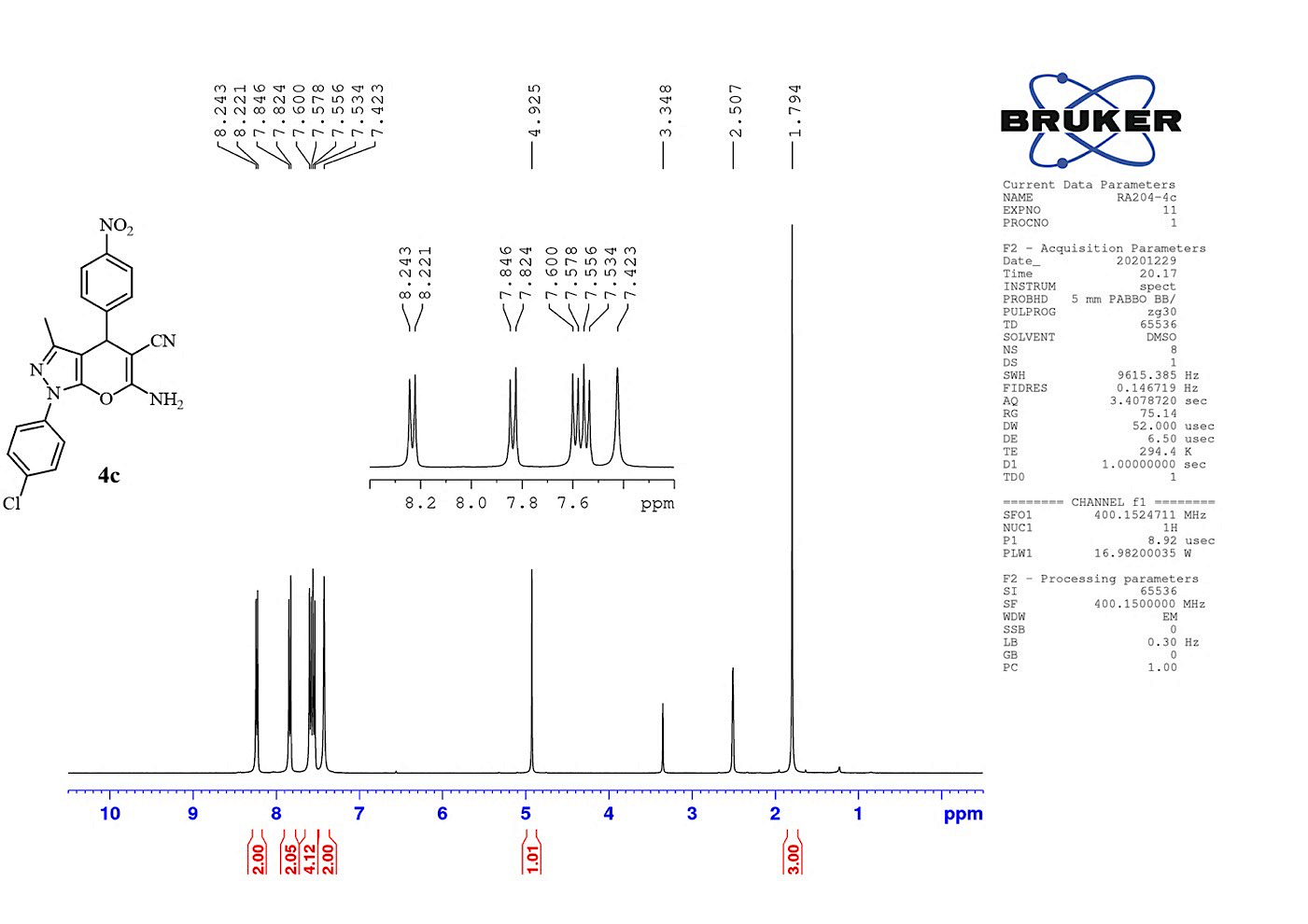
**

**Figure S5.** ^1^H NMR spectrum of compound **4c** at 400 MHz in DMSO-d_6_

_
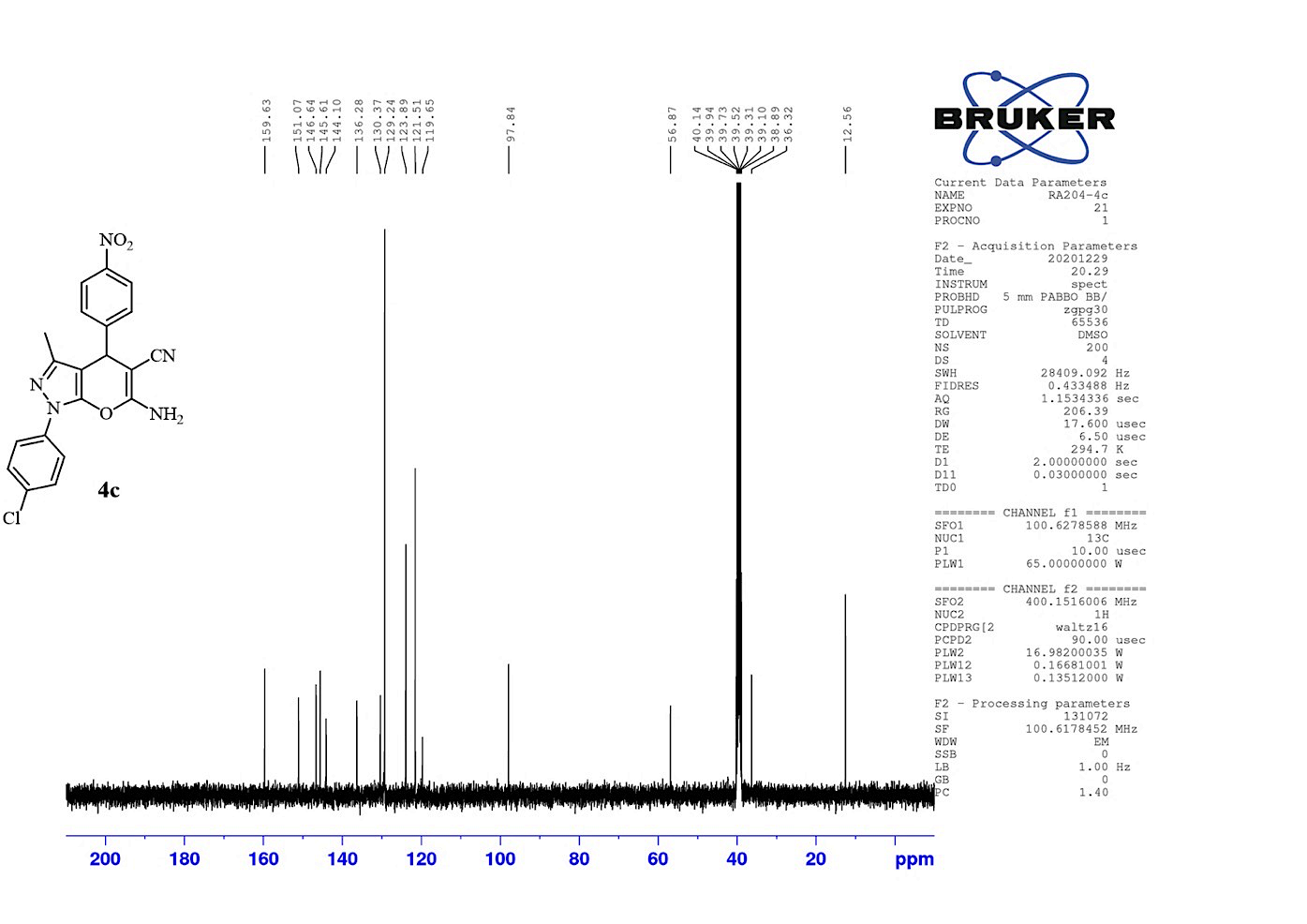
_

**Figure S6.** ^13^C{^1^H} NMR spectrum of compound **4c** at 100 MHz in DMSO-d_6_

_
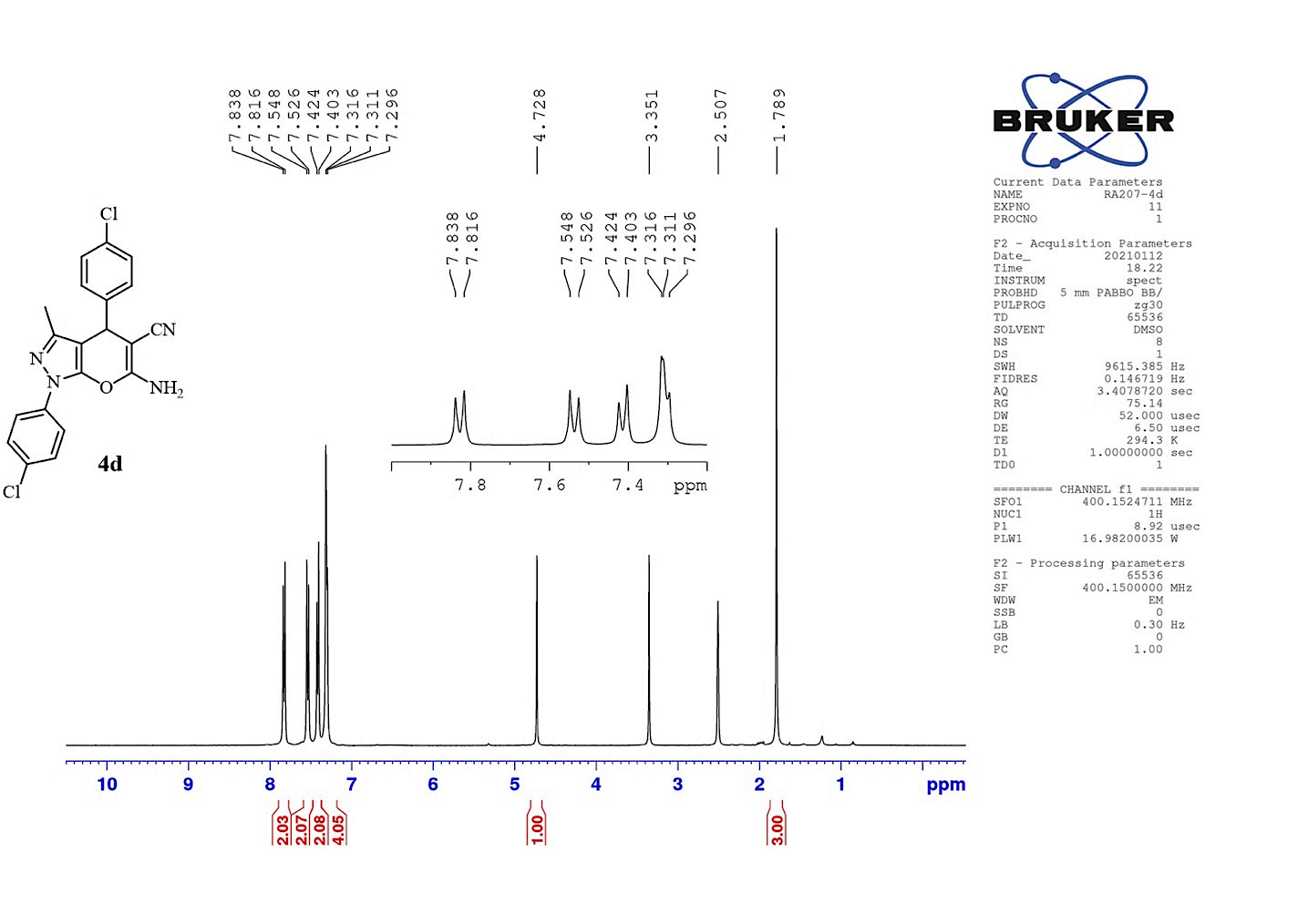
_**Figure S7.** ^1^H NMR spectrum of compound **4d** at 400 MHz in DMSO-d_6_

_
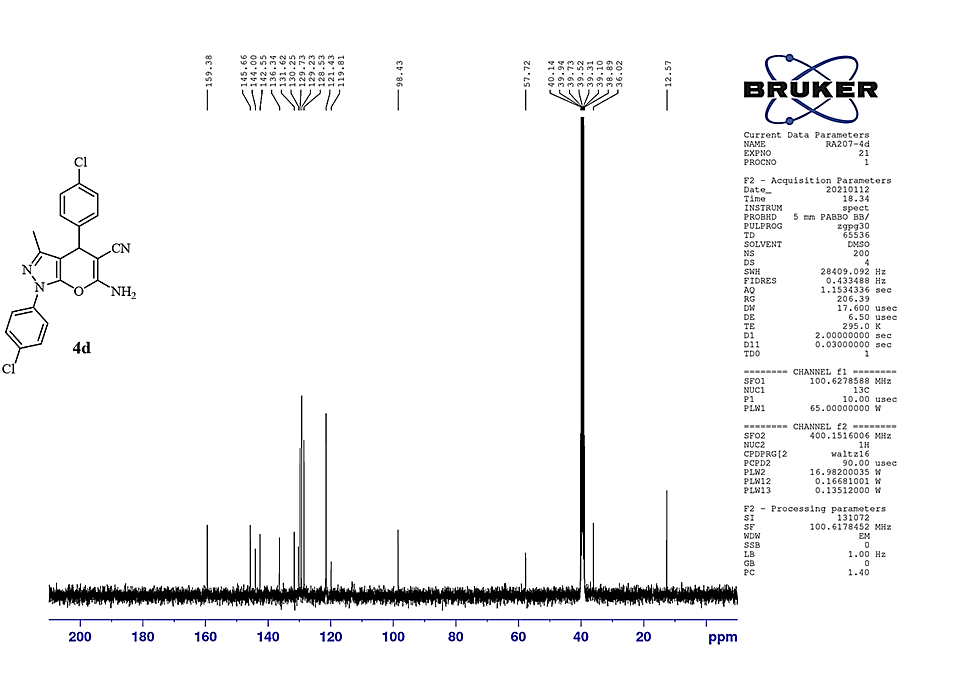
_

**Figure S8.** ^13^C{^1^H} NMR spectrum of compound **4d** at 100 MHz in DMSO-d_6_

**
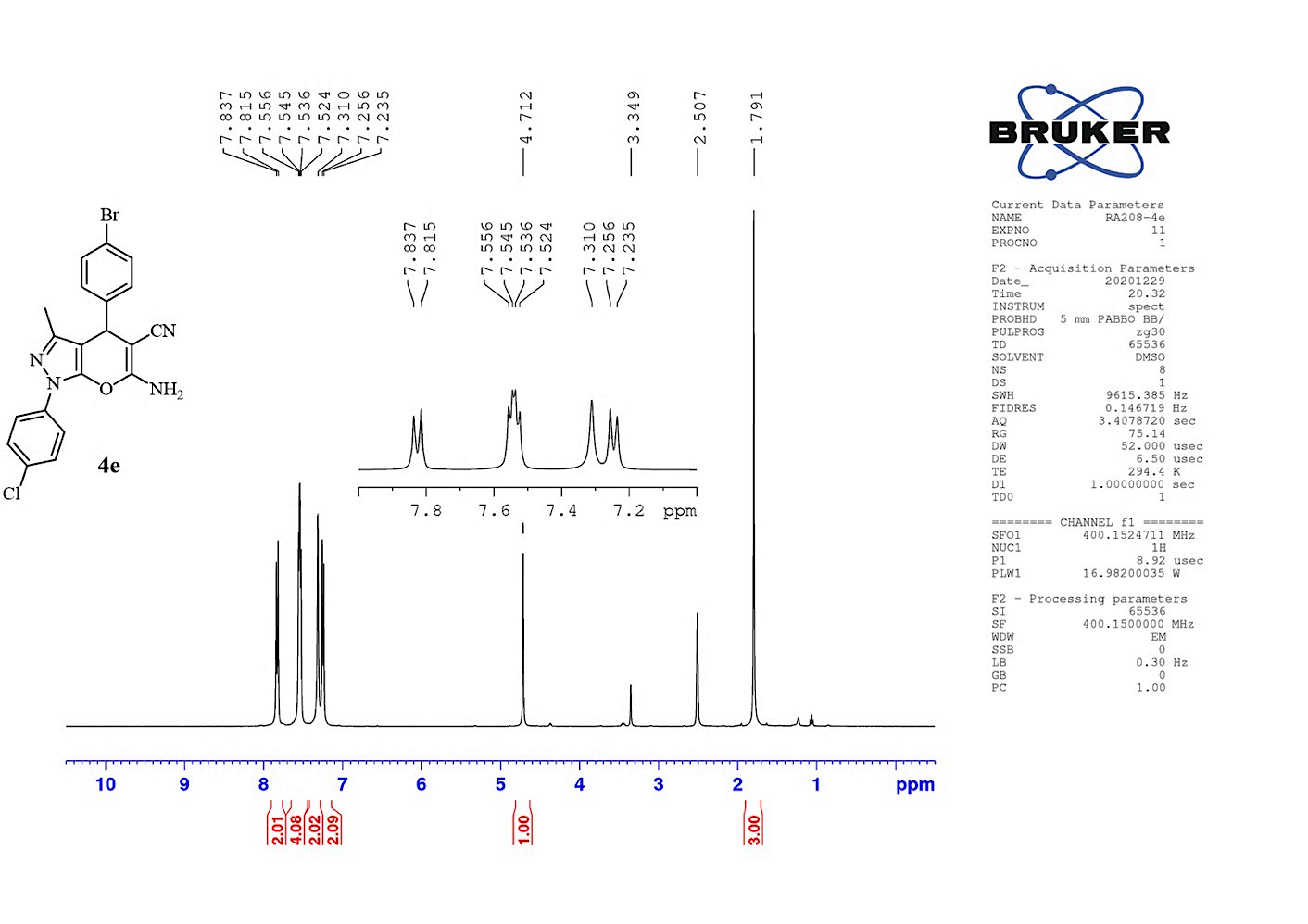
**

**Figure S9.** ^1^H NMR spectrum of compound **4e** at 400 MHz in DMSO-d_6_

_
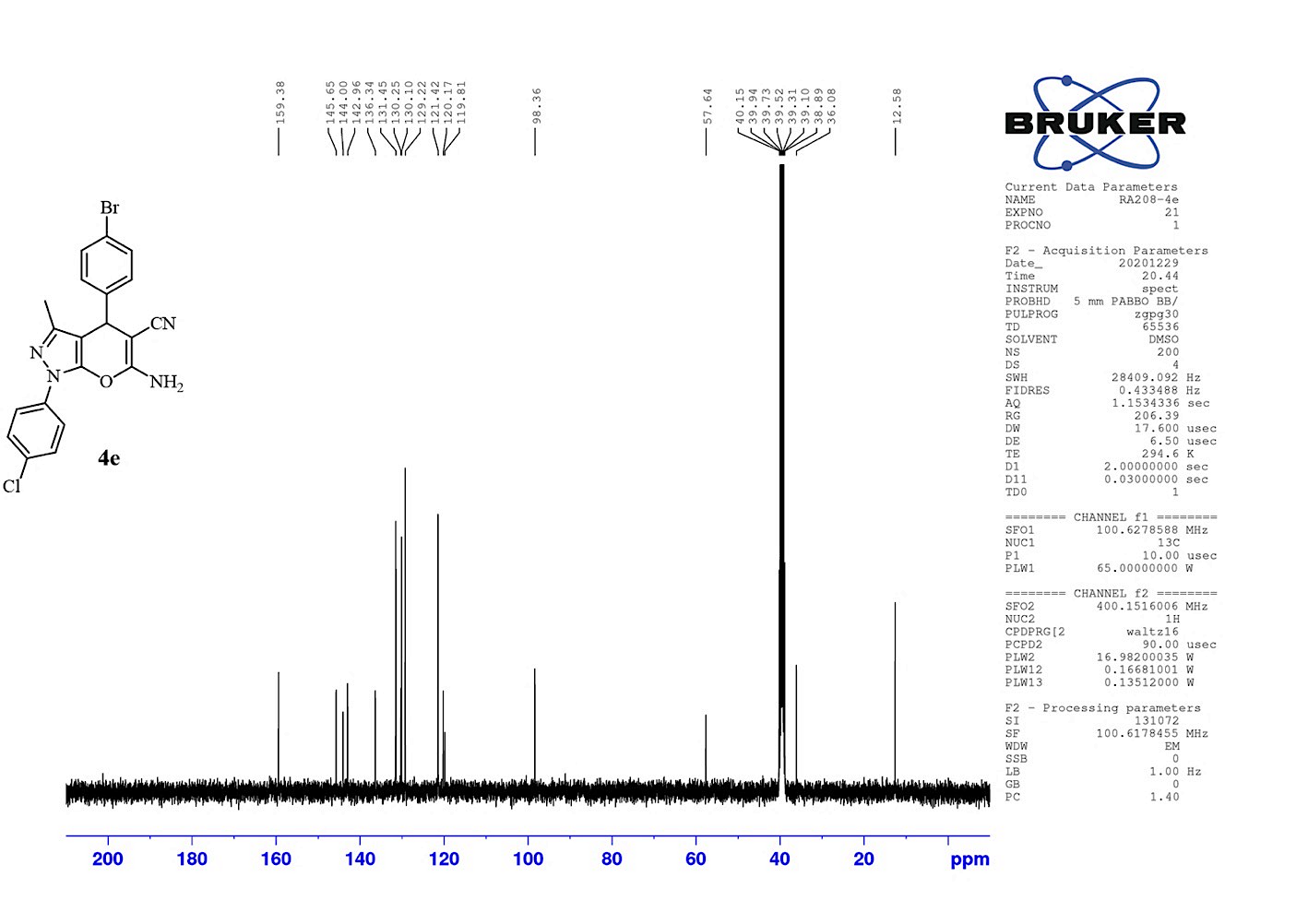
_

**Figure S10.** ^13^C{^1^H} NMR spectrum of compound **4e** at 100 MHz in DMSO-d_6_

**
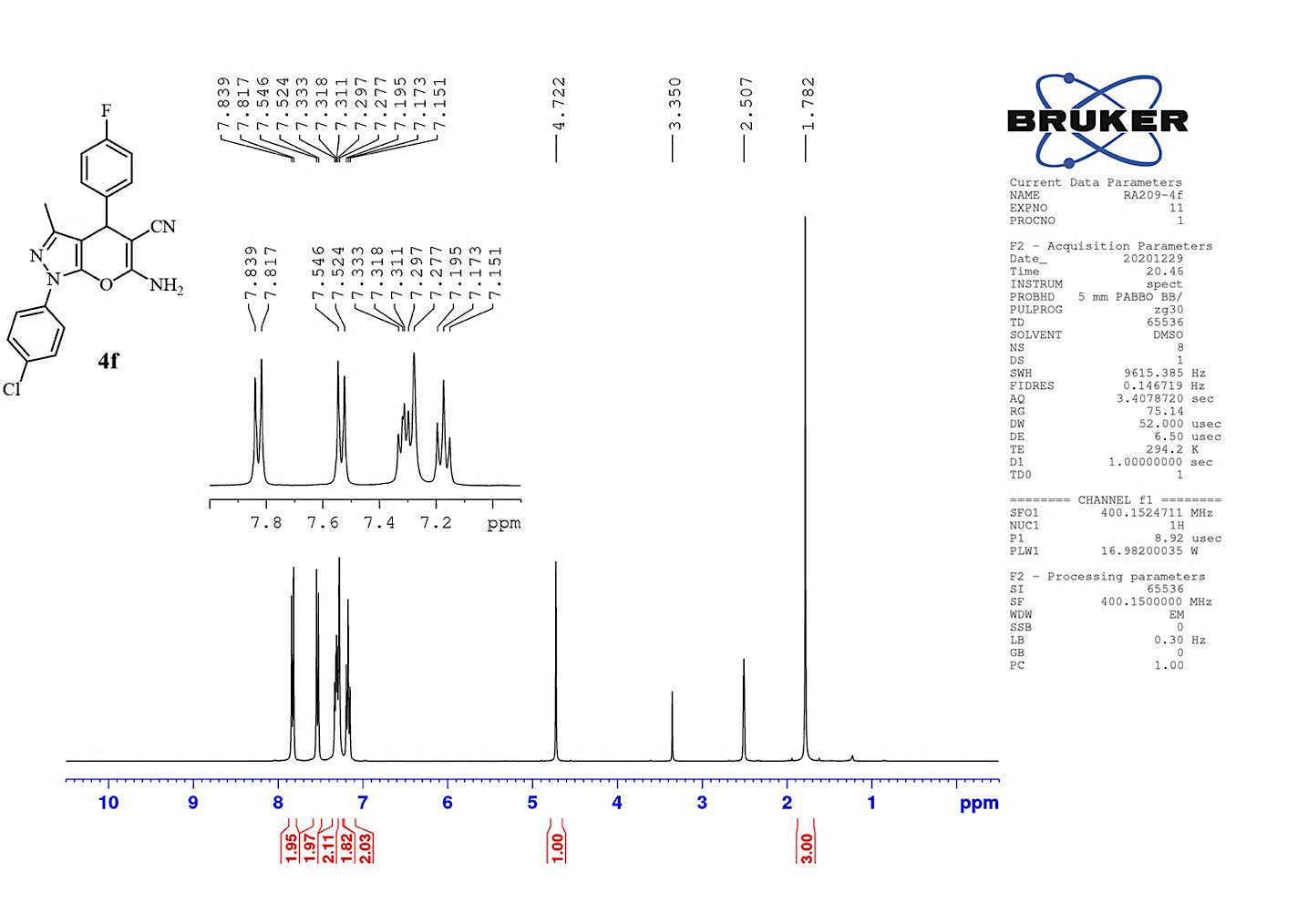
Figure S11.** ^1^H NMR spectrum of compound **4f** at 400 MHz in DMSO-d_6_

_
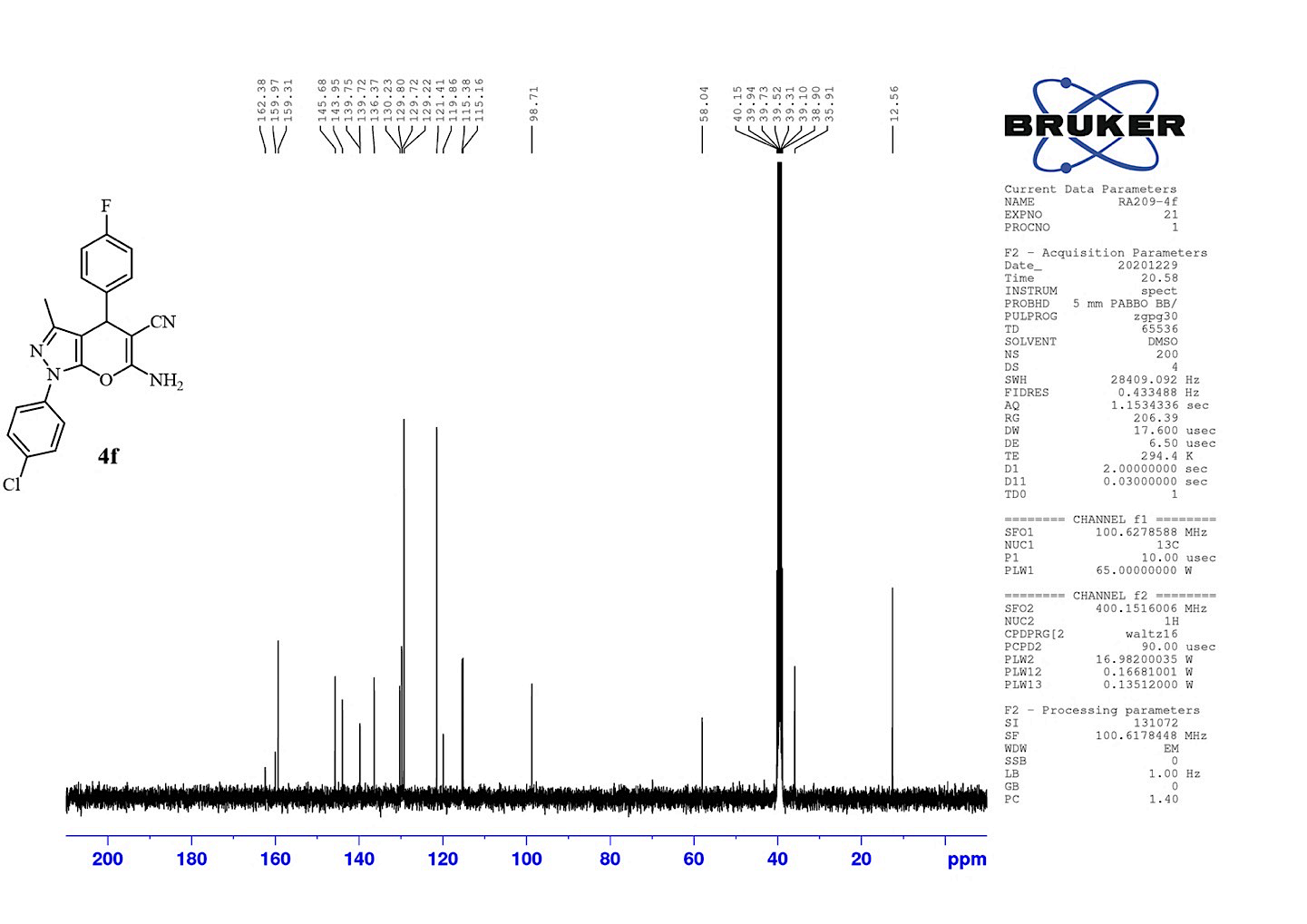
_

**Figure S12.** ^13^C{^1^H} NMR spectrum of compound **4f** at 100 MHz in DMSO-d_6_


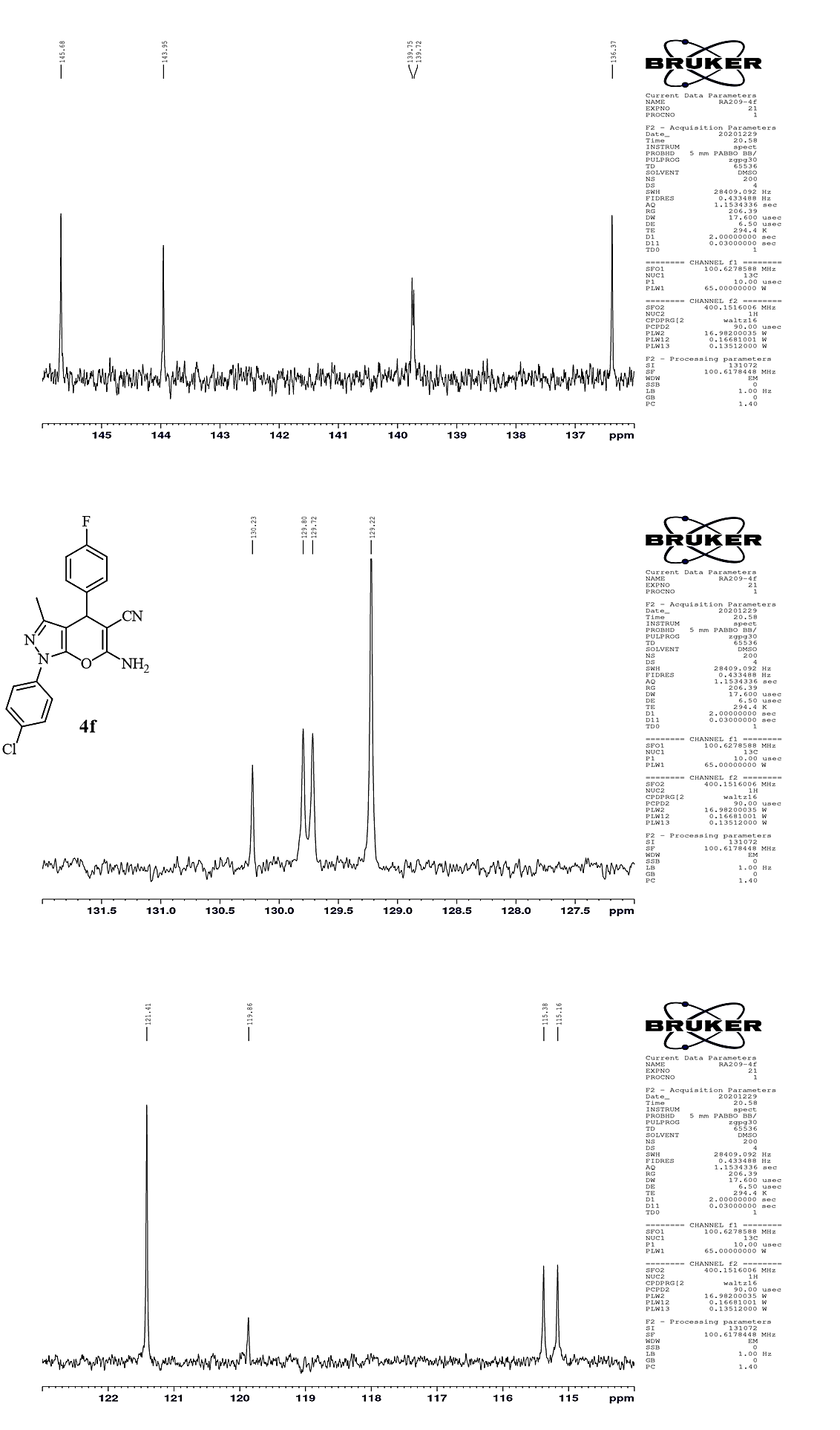


**Figure S13.** ^13^C{^1^H} NMR spectrum of compound **4f** at 100 MHz in DMSO-d_6_

**
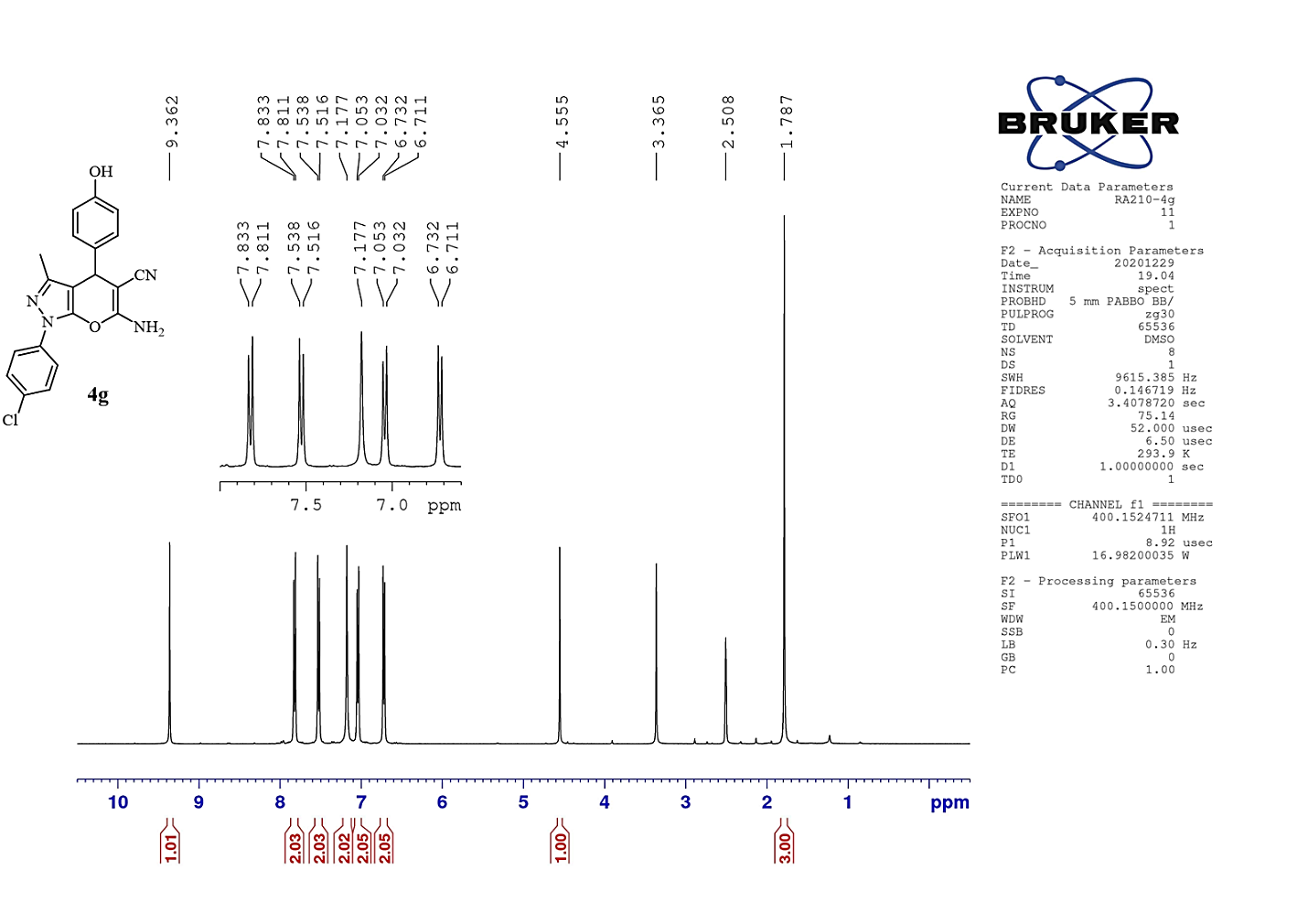
Figure S14.** ^1^H NMR spectrum of compound **4g** at 400 MHz in DMSO-d_6_

_
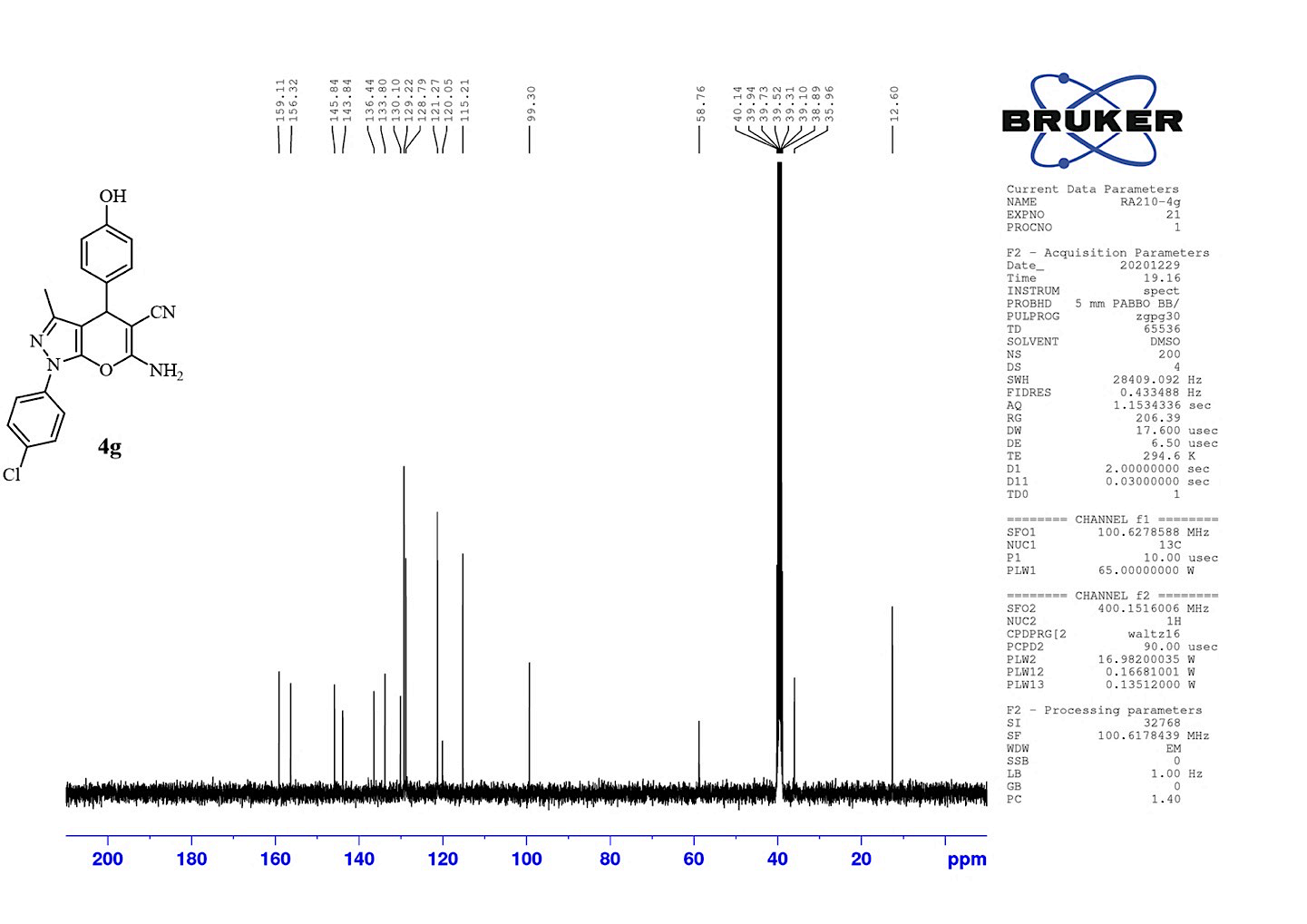
_

**Figure S15.** ^13^C{^1^H} NMR spectrum of compound **4g** at 100 MHz in DMSO-d_6_


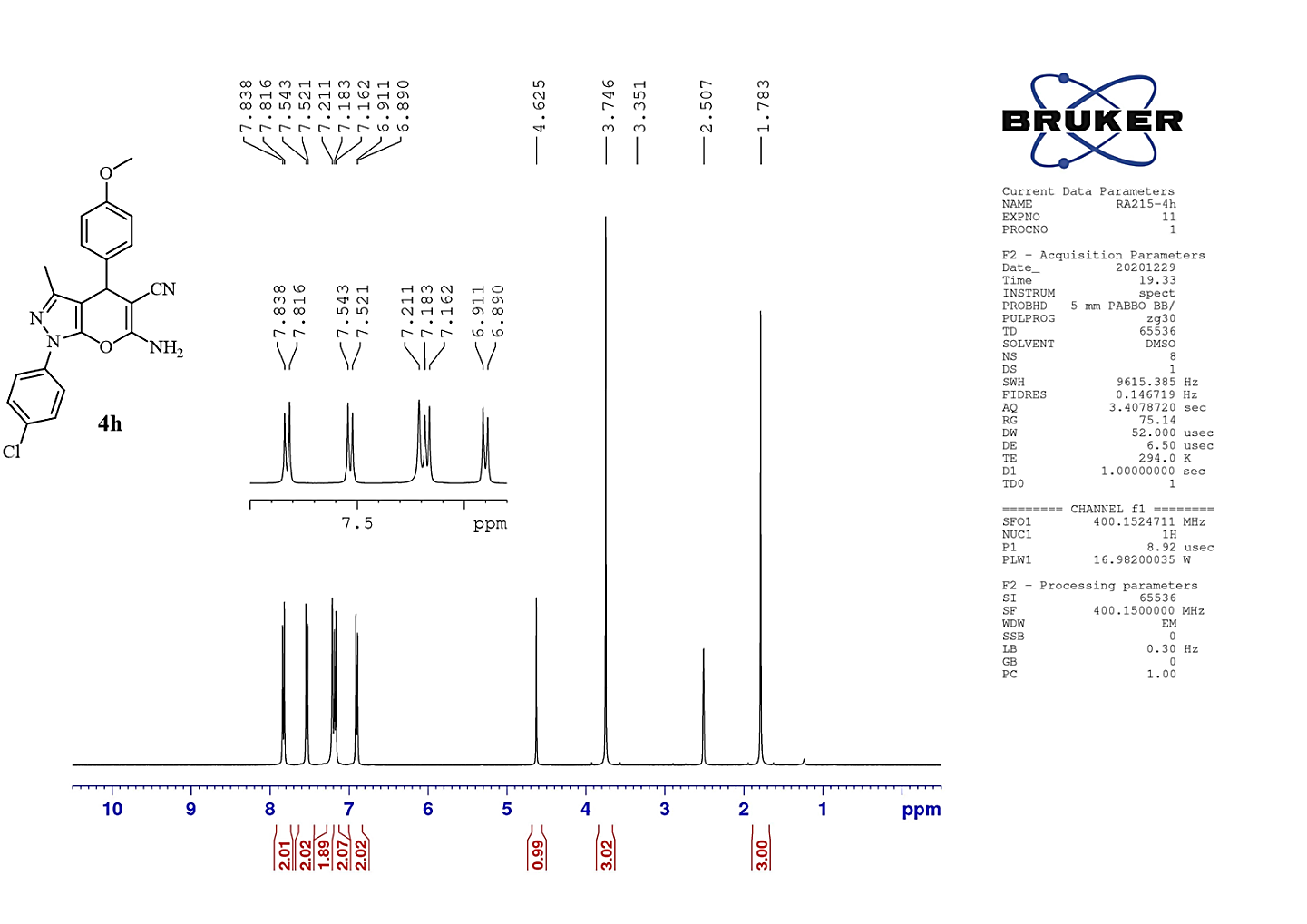


**Figure S16.** ^1^H NMR spectrum of compound **4h** at 400 MHz in DMSO-d_6_

_
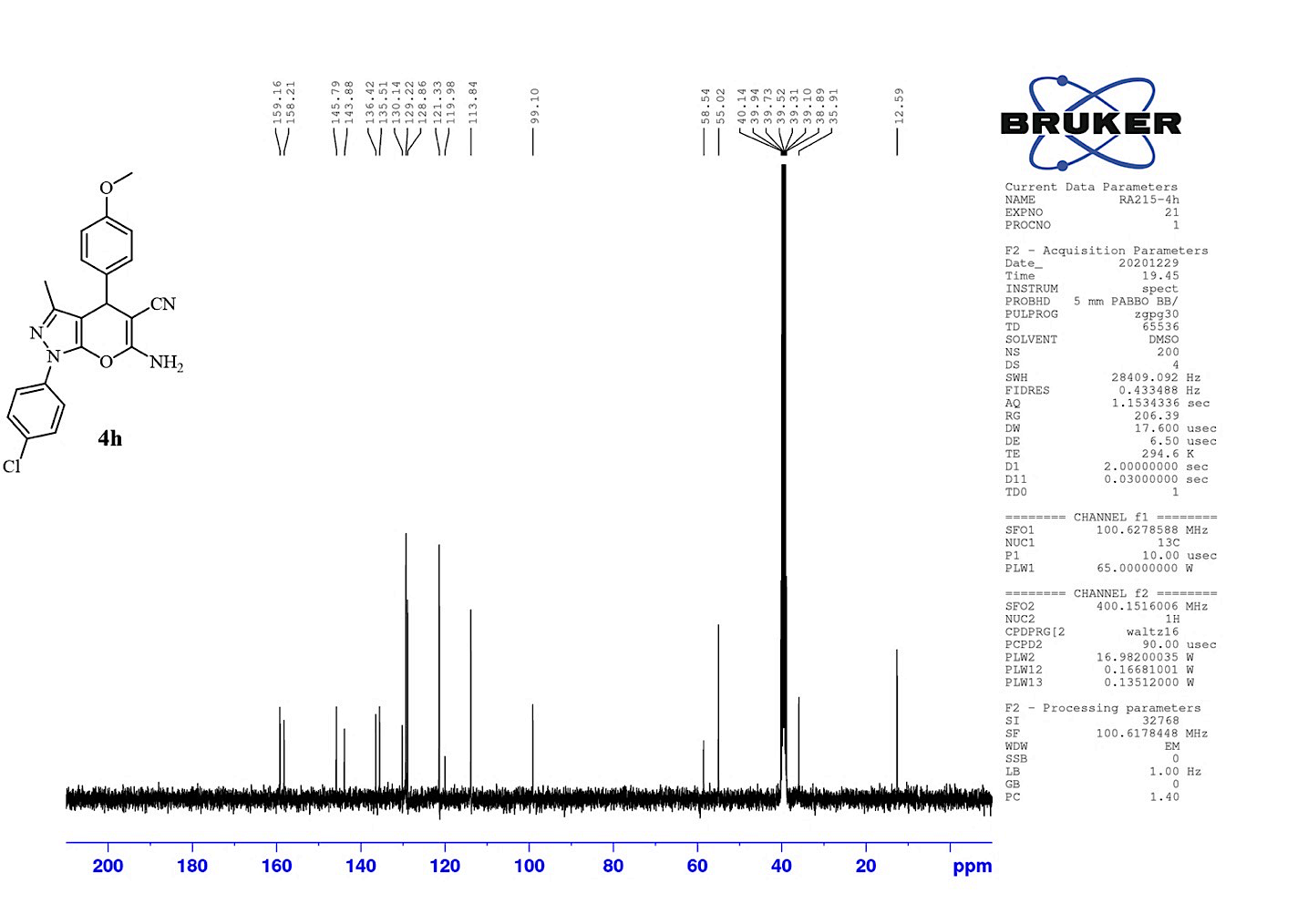
_

**Figure S17.** ^13^C{^1^H} NMR spectrum of compound **4h** at 100 MHz in DMSO-d_6_


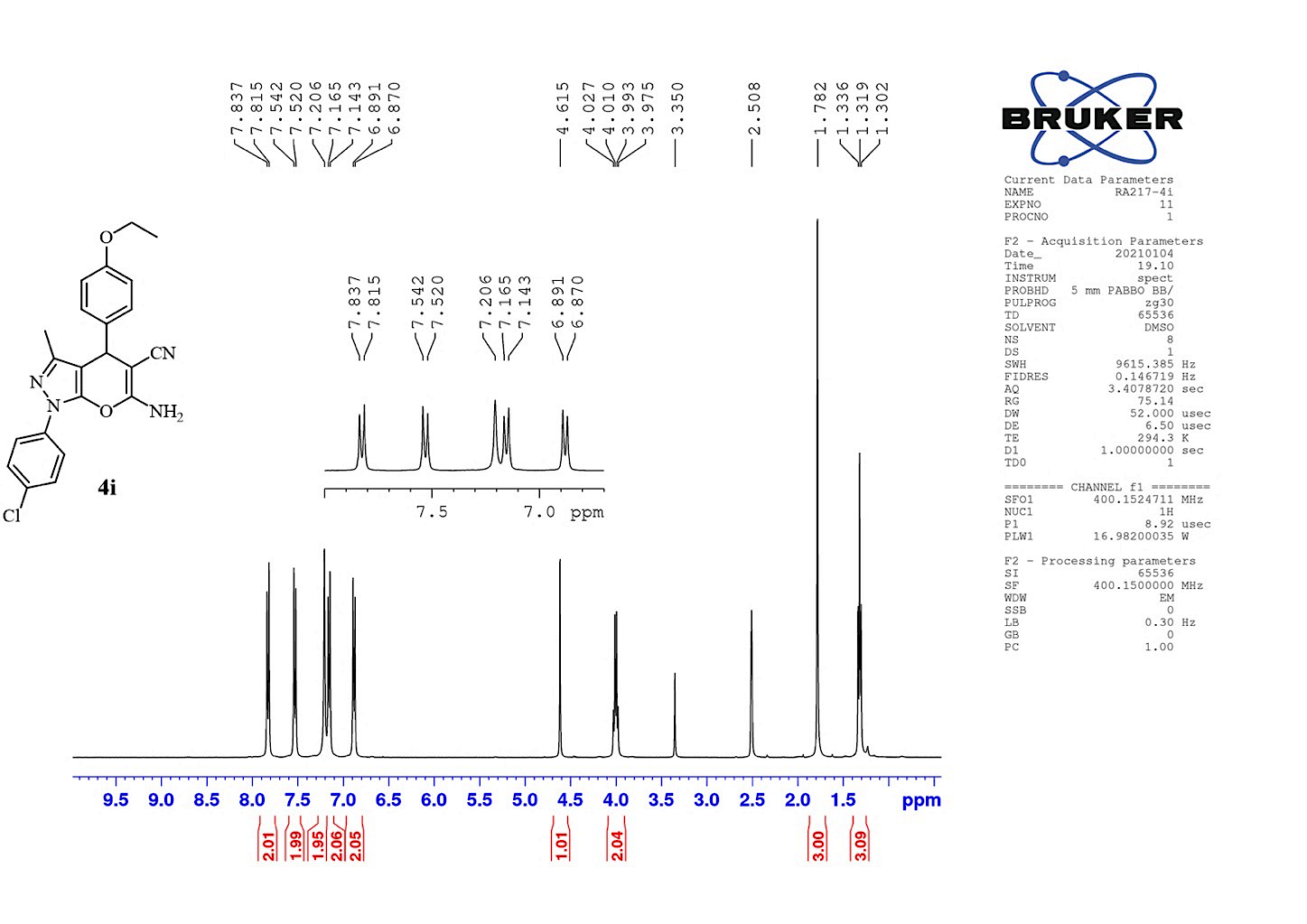


**Figure S18.** ^1^H NMR spectrum of compound **4i** at 400 MHz in DMSO-d_6_

_
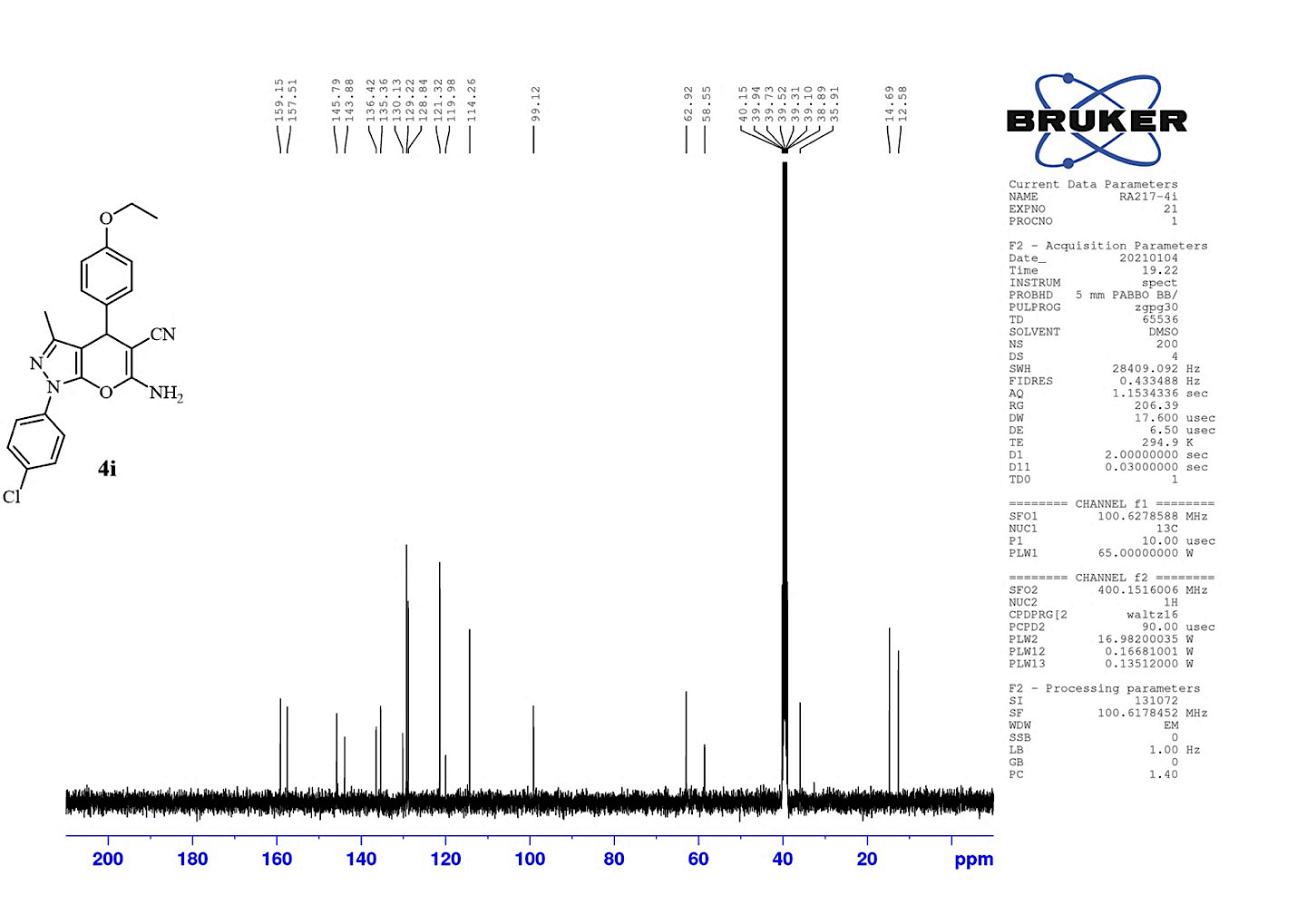
_

**Figure S19.** ^13^C{^1^H} NMR spectrum of compound **4i** at 100 MHz in DMSO-d_6_

**
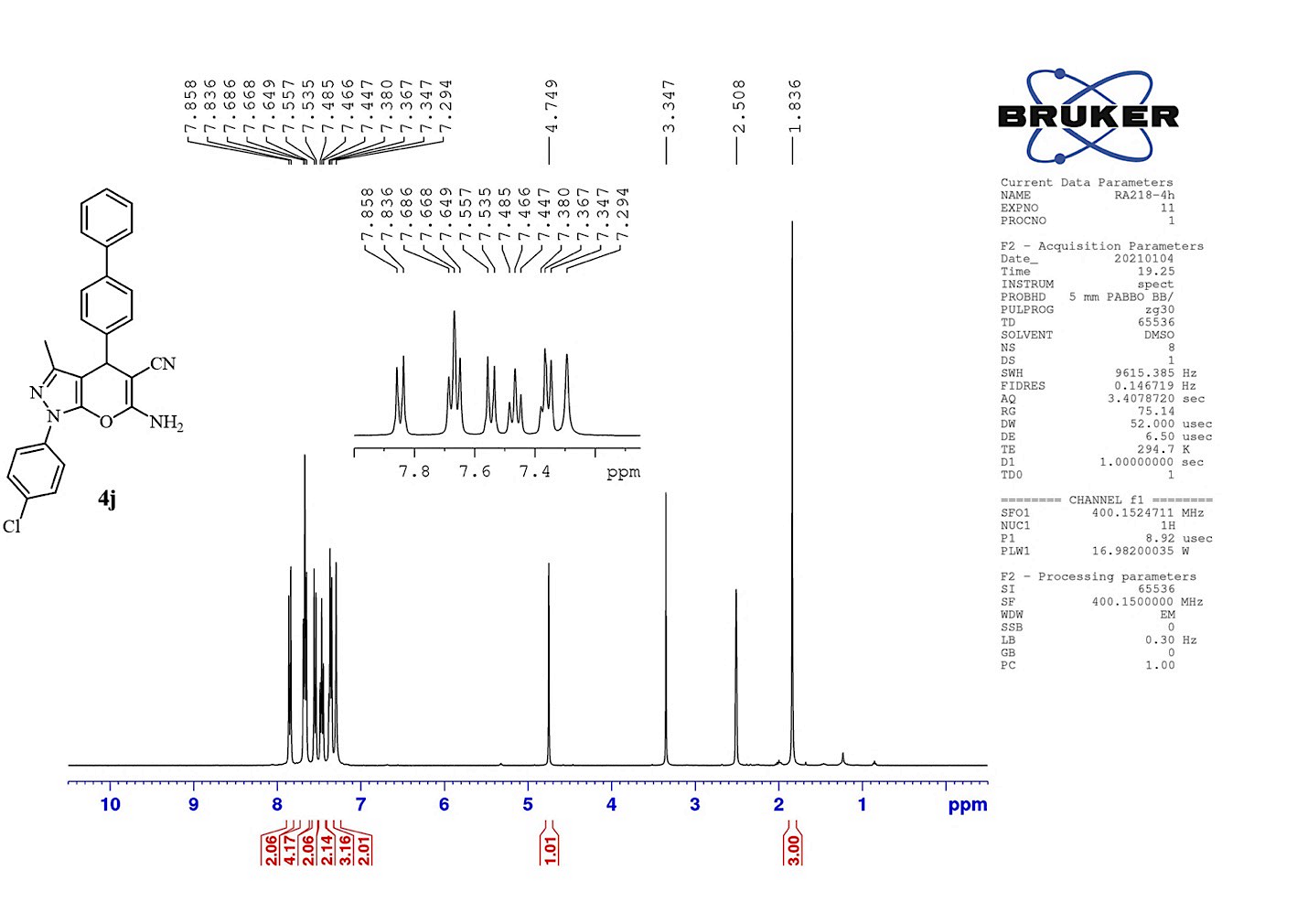
Figure S20.** ^1^H NMR spectrum of compound **4j** at 400 MHz in DMSO-d_6_

_
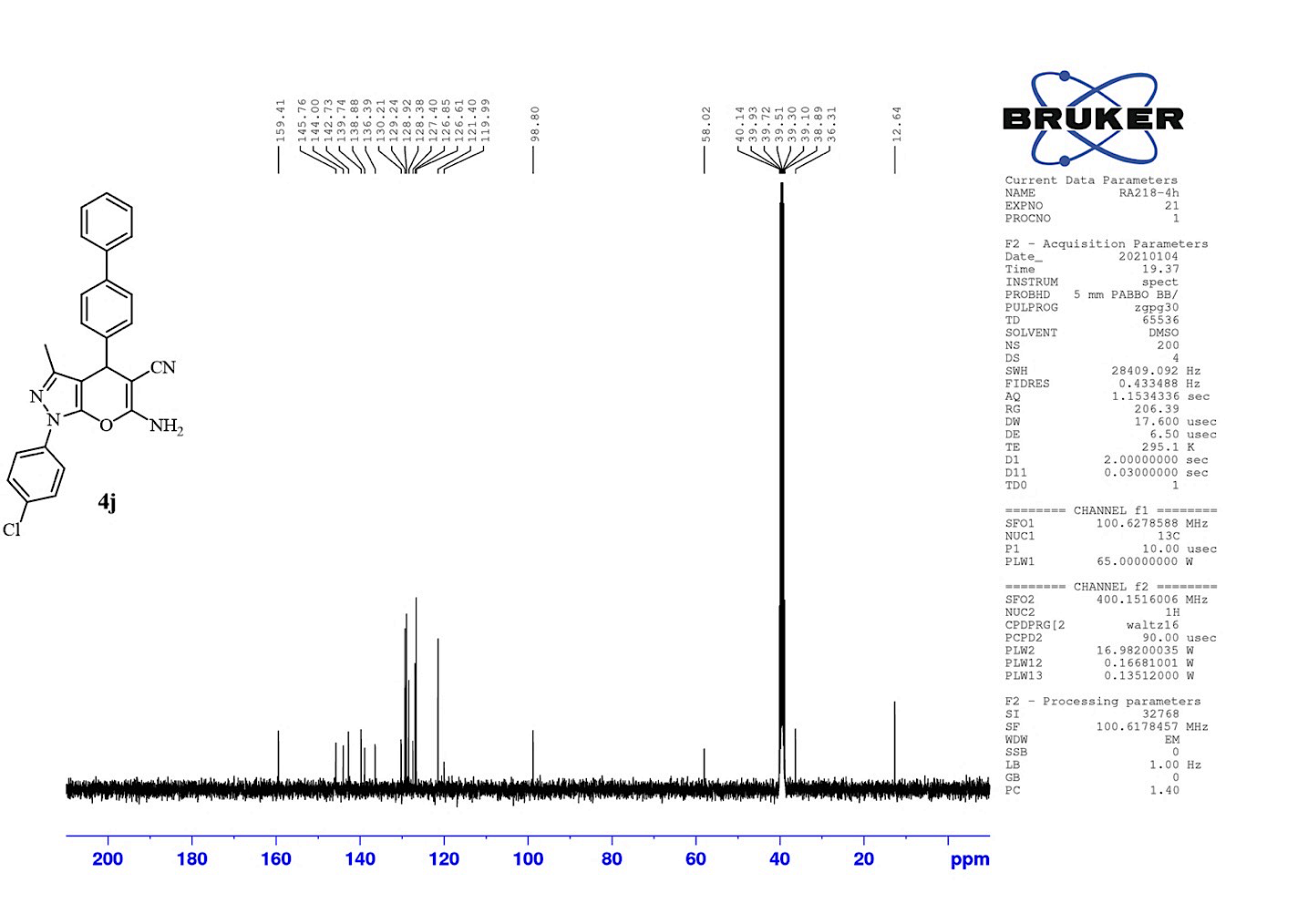
_

**Figure S21.** ^13^C{^1^H} NMR spectrum of compound **4j** at 100 MHz in DMSO-d_6_

1. Copies of HRMS of **4a**-**4j**


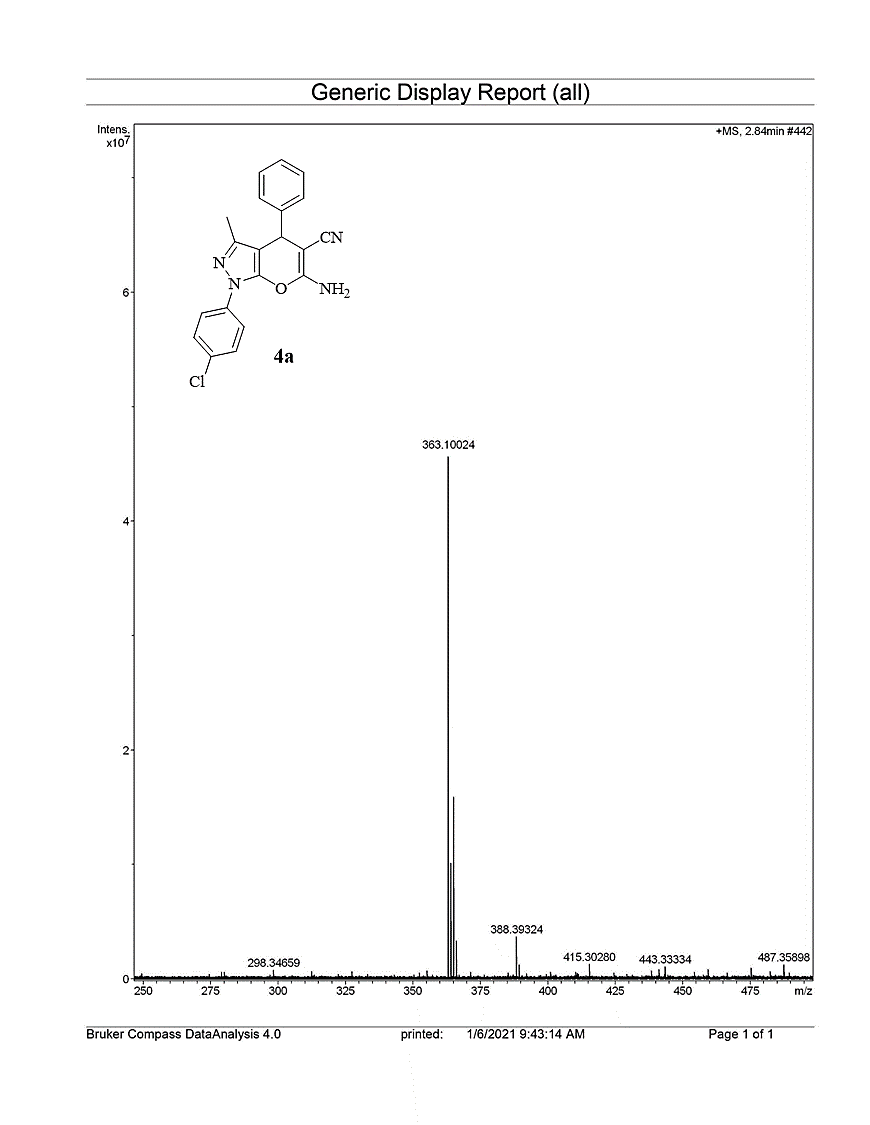
**Figure S22.** HRMS of compound **4a**

**
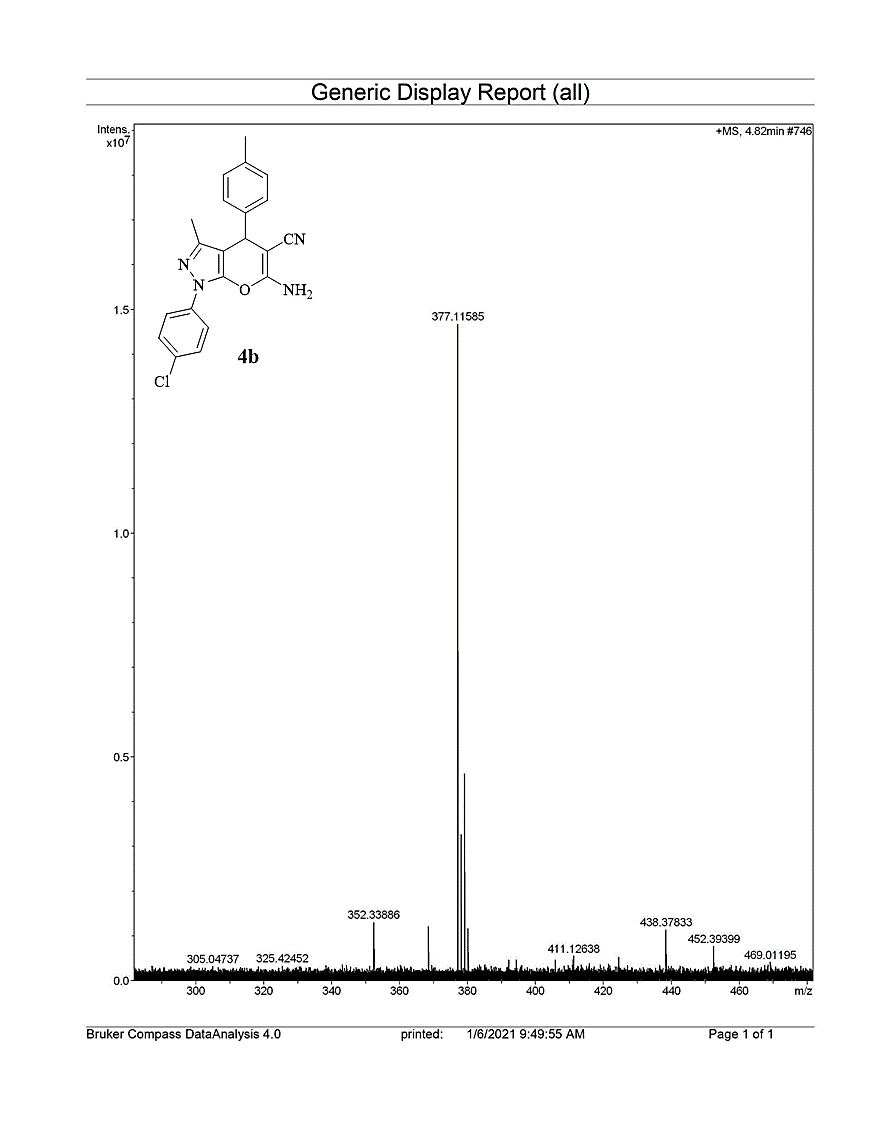
**

**Figure S23.** HRMS of compound **4b**

**
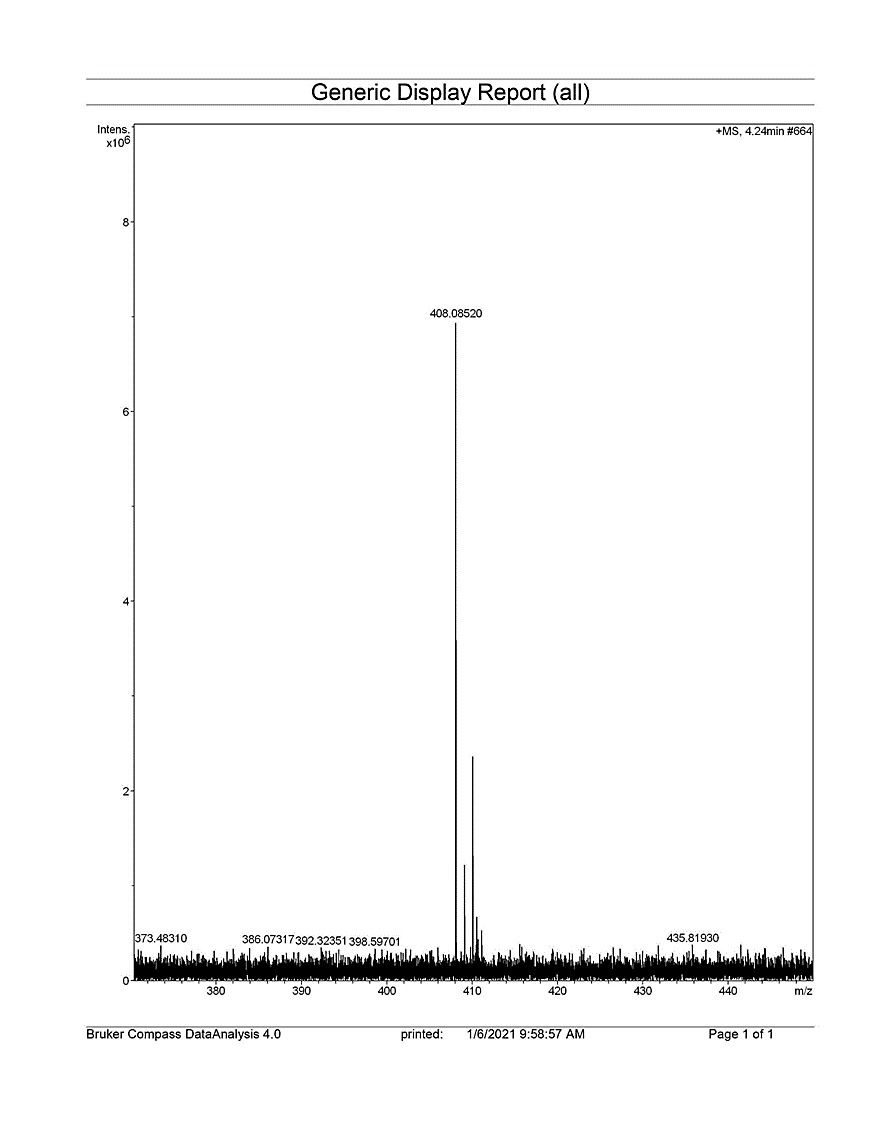
**

**Figure S24.** HRMS of compound **4c**

**
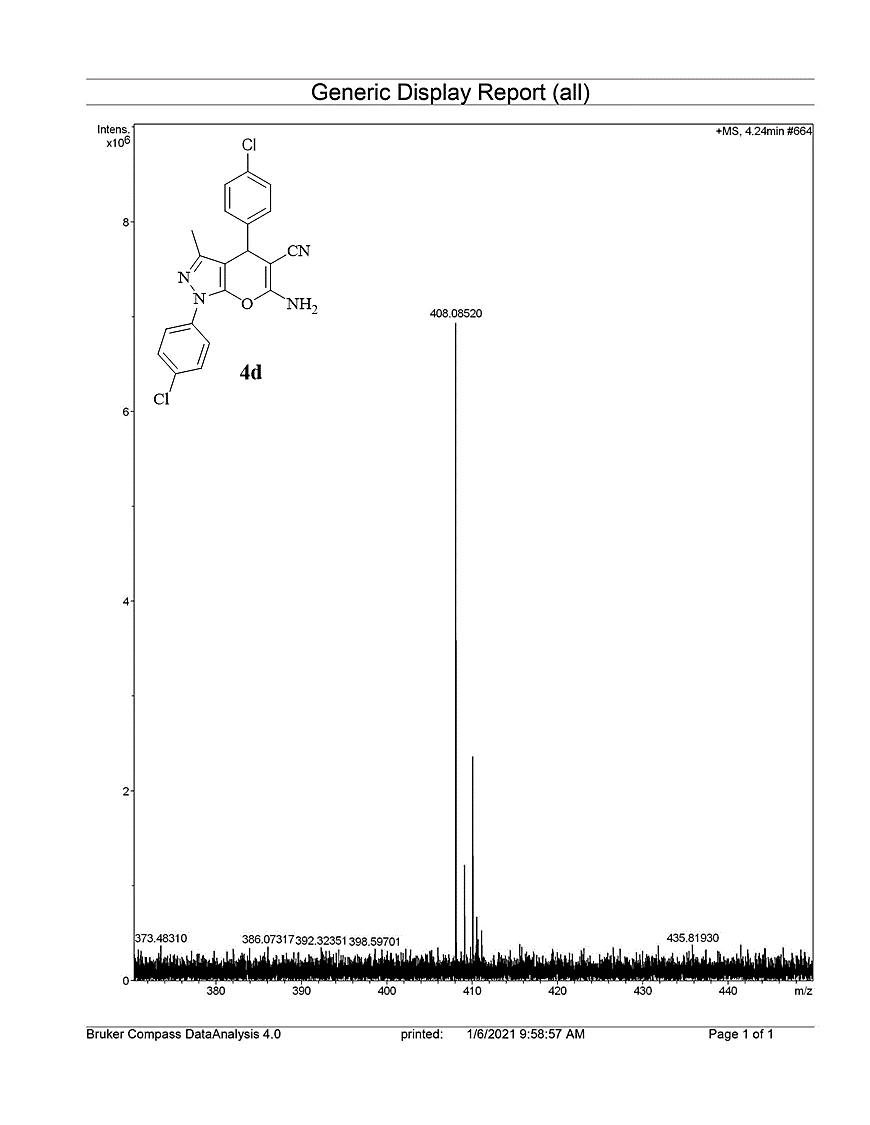
**

**Figure S25.** HRMS of compound **4d**

**
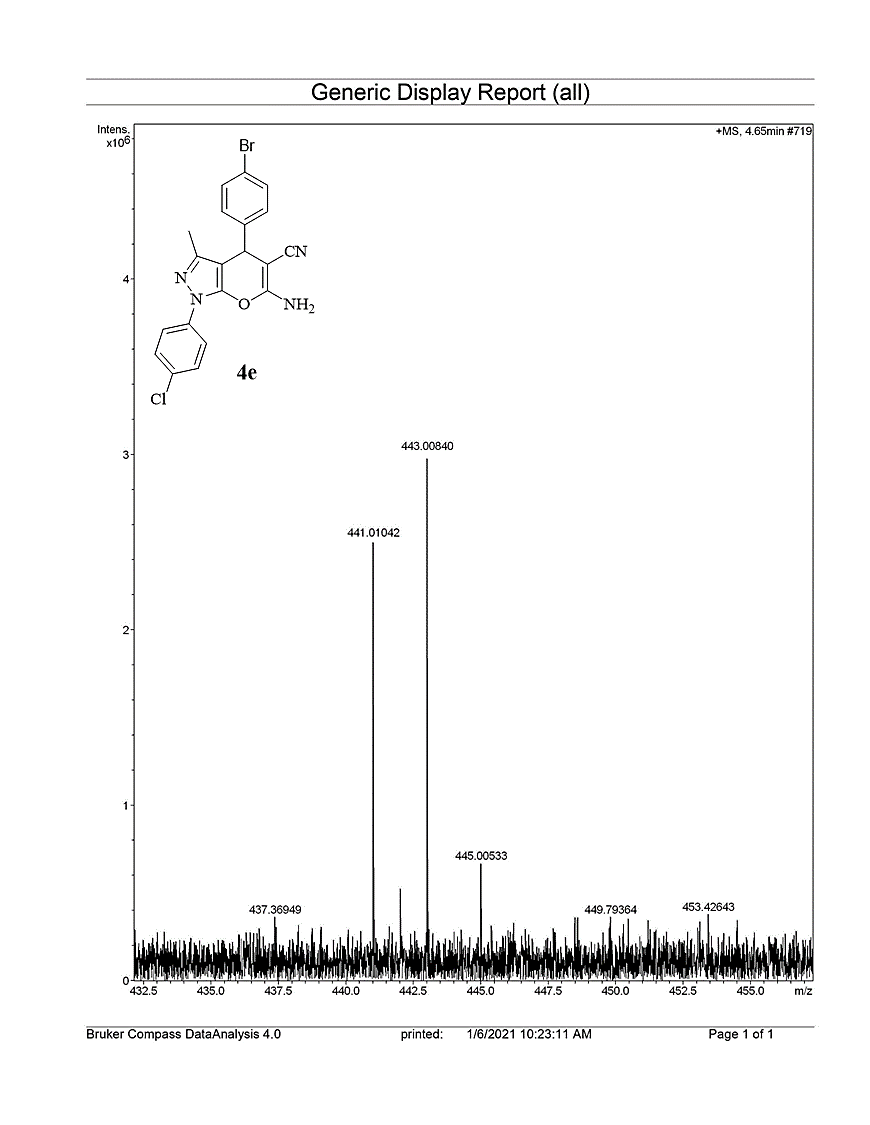
**

**Figure S26.** HRMS of compound **4e**

**
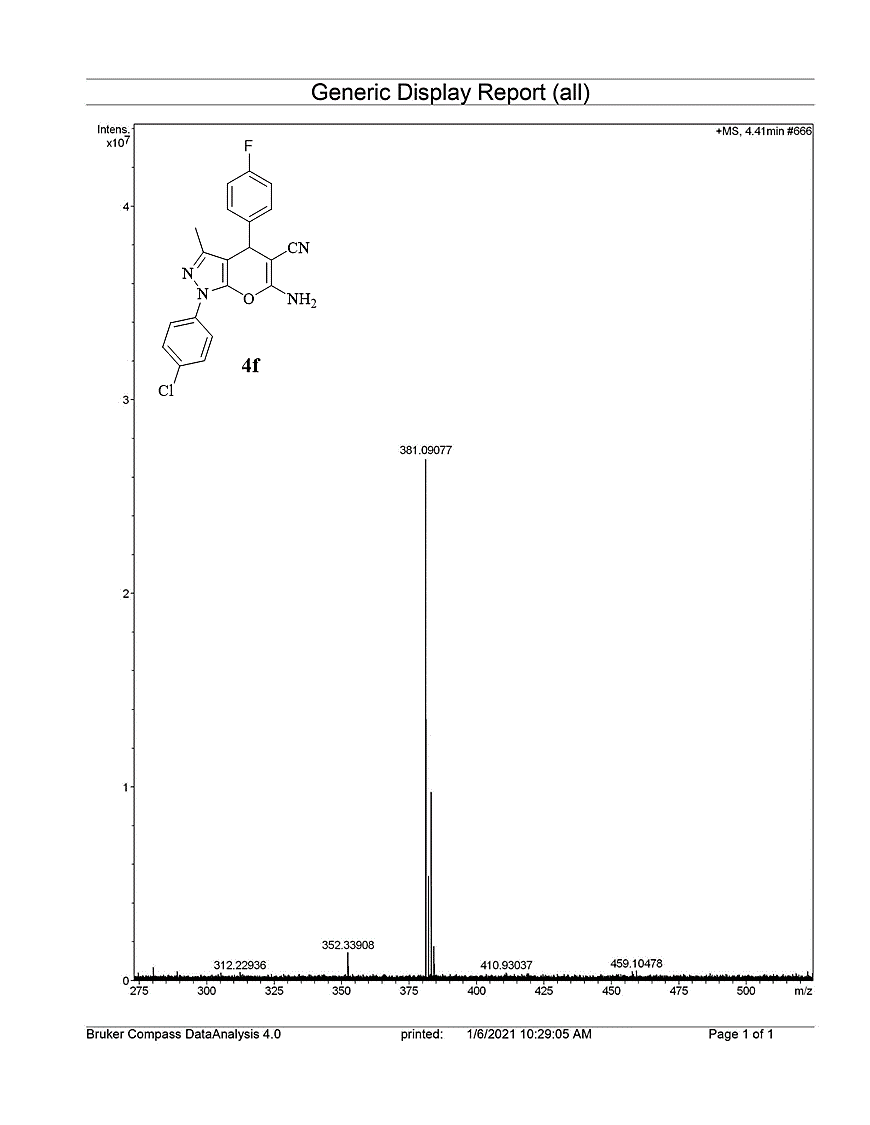
**

**Figure S27.** HRMS of compound **4f**

**
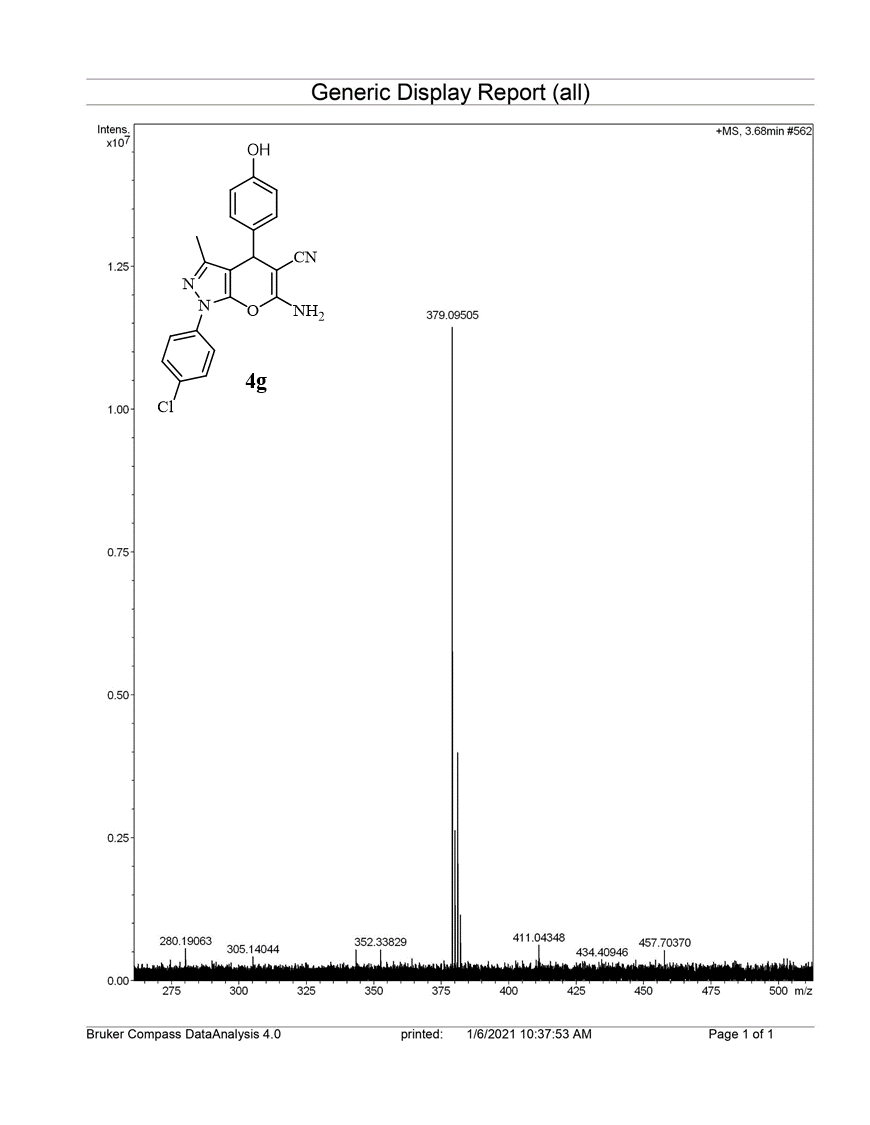
**

**Figure S28.** HRMS of compound **4g**

**
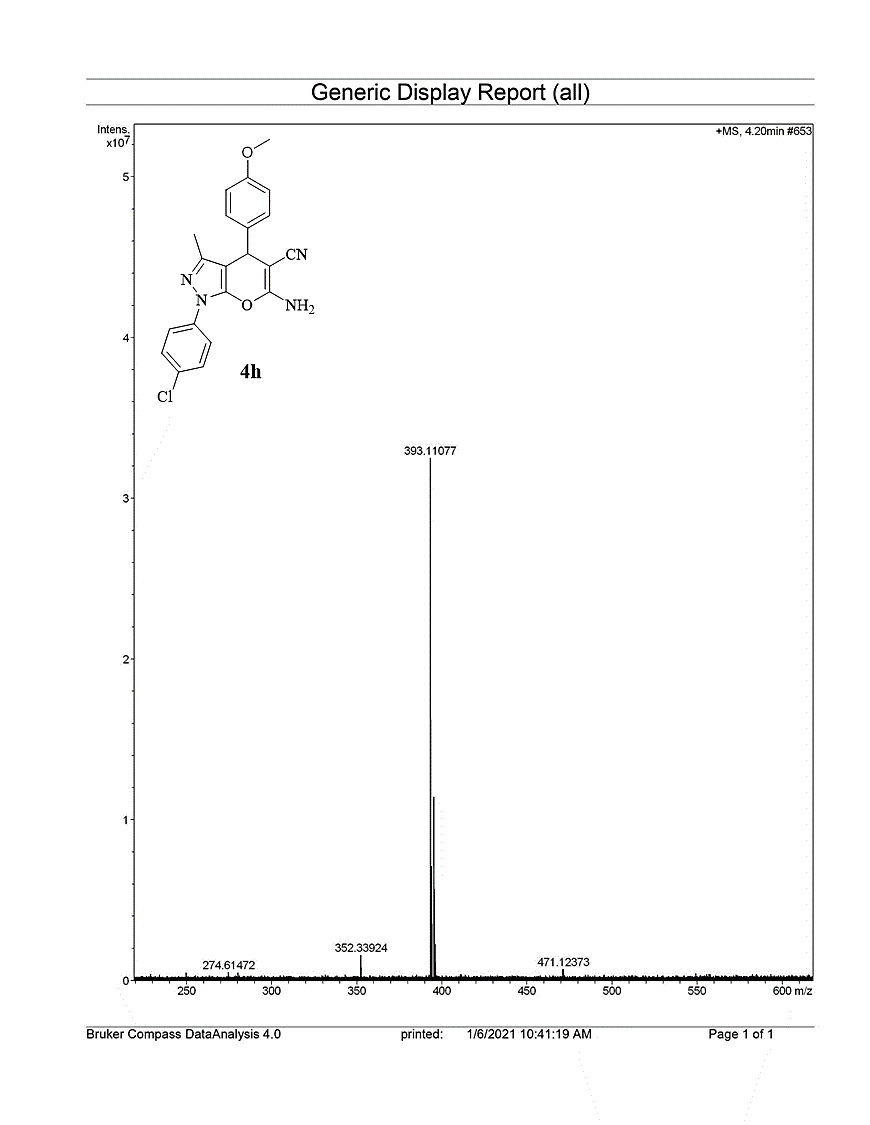
**

**Figure S29.** HRMS of compound **4h**

**
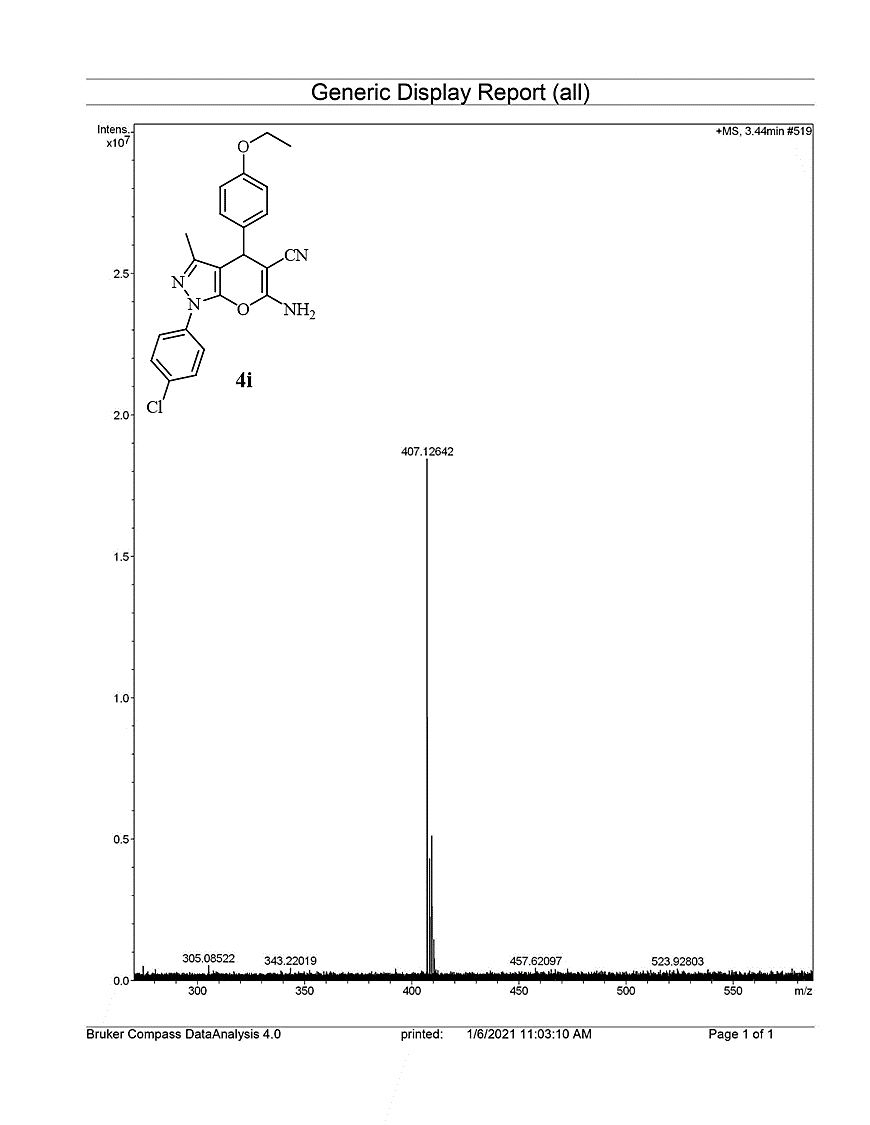
**

**Figure S30.** HRMS of compound **4i**

_
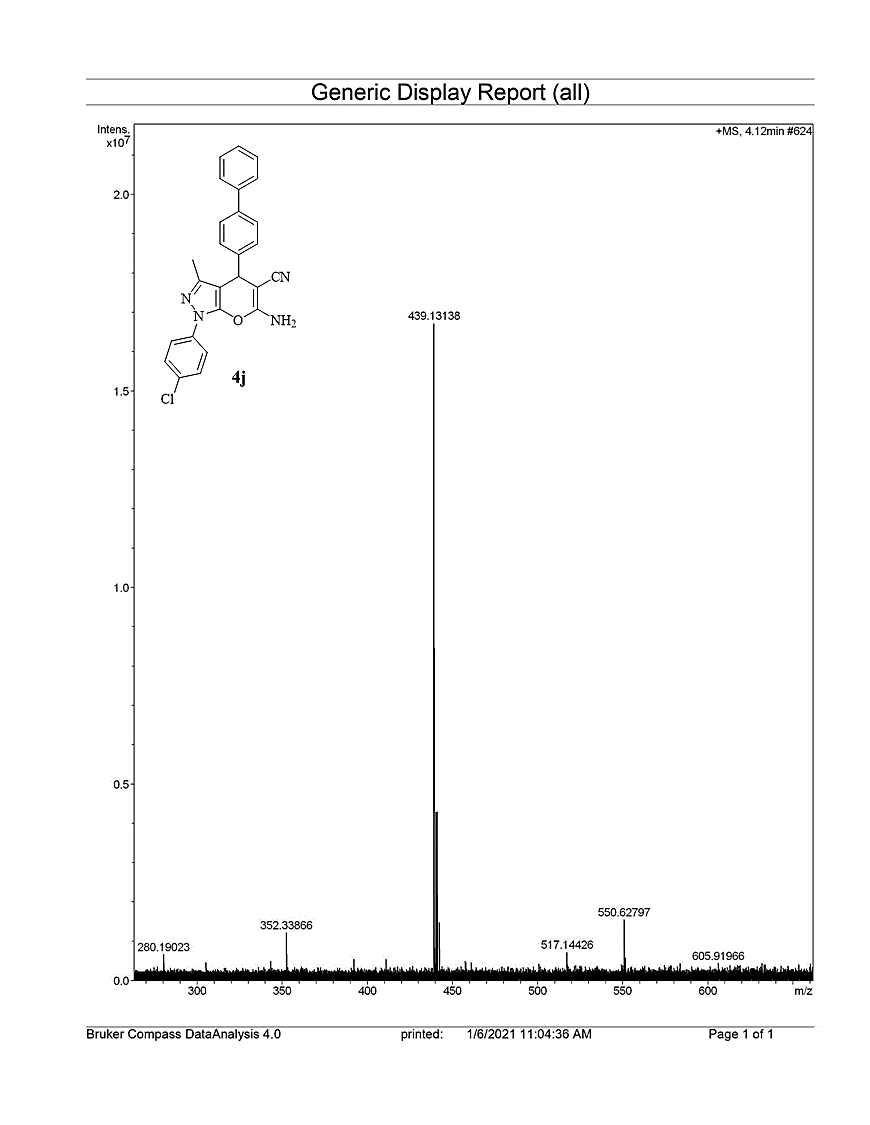
_

**Figure S31.** HRMS of compound **4j**
